# Physical literacy levels of Canadian children aged 8–12 years: descriptive and normative results from the RBC Learn to Play–CAPL project

**DOI:** 10.1186/s12889-018-5891-x

**Published:** 2018-10-02

**Authors:** Mark S. Tremblay, Patricia E. Longmuir, Joel D. Barnes, Kevin Belanger, Kristal D. Anderson, Brenda Bruner, Jennifer L. Copeland, Christine Delisle Nyström, Melanie J. Gregg, Nathan Hall, Angela M. Kolen, Kirstin N. Lane, Barbi Law, Dany J. MacDonald, Luc J. Martin, Travis J. Saunders, Dwayne Sheehan, Michelle R. Stone, Sarah J. Woodruff

**Affiliations:** 1 Healthy Active Living and Obesity Research Group, Children’s Hospital of Eastern Ontario Research Institute, 401 Smyth Road, Ottawa, ON K1H 8L1 Canada; 2 Centre for Sport and Exercise Education, Camosun College, Victoria, BC V9E 2C1 Canada; 3 School of Physical and Health Education, Nipissing University, North Bay, ON P1B 8L7 Canada; 4 Department of Kinesiology and Physical Education, University of Lethbridge, Lethbridge, AB T1K 3M4 Canada; 5 Department of Kinesiology and Applied Health, University of Winnipeg, Winnipeg, MB R3B 2E9 Canada; 6 Department of Human Kinetics, St. Francis Xavier University, Antigonish, NS B2G 2W5 Canada; 7 Department of Applied Human Sciences, University of Prince Edward Island, Charlottetown, PEI C1A 4P3 Canada; 8 School of Kinesiology and Health Studies, Queen’s University, Kingston, ON K7L 3N6 Canada; 9 Department of Health and Physical Education, Mount Royal University, Calgary, AB T3E 6K6 Canada; 10 School of Health and Human Performance, Dalhousie University, Halifax, NS B3H 4R2 Canada; 11 Department of Kinesiology, University of Windsor, Windsor, ON N9B 3P4 Canada

**Keywords:** Daily behaviour, Physical competence, Knowledge, Understanding, Motivation, Confidence, Physical fitness, Motor skill, Physical activity

## Abstract

**Background:**

The current physical literacy level of Canadian children is unknown. The Royal Bank of Canada (RBC) Learn to Play – Canadian Assessment of Physical Literacy (CAPL) project, which is anchored in the Canadian consensus statement definition of physical literacy, aimed to help establish the current physical literacy level of Canadian children.

**Methods:**

The CAPL was used to assess the physical literacy (and component domains: Daily Behaviour, Physical Competence, Knowledge and Understanding, and Motivation and Confidence) of Canadian children aged 8–12 years. Data were collected from 11 sites across Canada, yielding a sample of 10,034 participants (5030 girls). Descriptive statistics by age and gender were calculated and percentile distributions of physical literacy scores, including each domain and individual measure, were derived.

**Results:**

The mean age of participants was 10.1 ± 1.2 years. Total physical literacy scores (out of 100) were on average 63.1 ± 13.0 for boys and 62.2 ± 11.3 for girls. For boys and girls respectively, domain scores were 19.9 ± 4.7 and 19.3 ± 4.1 (out of 32) for Physical Competence; 18.6 ± 7.9 and 18.5 ± 7.4 (out of 32) for Daily Behaviour; 12.7 ± 2.8 and 12.2 ± 2.6 (out of 18) for Motivation and Confidence; and 11.8 ± 2.8 and 12.2 ± 2.6 (out of 18) for Knowledge and Understanding. Physical Competence measures were on average 28.1 ± 8.4 cm (sit-and-reach flexibility), 33.5 ± 9.4 kg (grip strength, right + left), 23.4 ± 14.1 laps (Progressive Aerobic Cardiovascular Endurance Run [PACER] shuttle run), 61.8 ± 43.8 s (isometric plank), 19.0 ± 3.8 kg/m^2^ (body mass index), 67.3 ± 10.8 cm (waist circumference), and 20.6 ± 3.9 out of 28 points for the Canadian Agility and Movement Skill Assessment (CAMSA), with scores for boys higher than girls and older children higher than younger children for grip strength, PACER, plank, and CAMSA score. Girls and younger children had better scores on the sit-and-reach flexibility than boys and older children. Daily pedometer step counts were higher in boys than girls (12,355 ± 4252 vs. 10,779 ± 3624), and decreased with age.

**Conclusions:**

These results provide the largest and most comprehensive assessment of physical literacy of Canadian children to date, providing a “state of the nation” baseline, and can be used to monitor changes and inform intervention strategies going forward.

**Electronic supplementary material:**

The online version of this article (10.1186/s12889-018-5891-x) contains supplementary material, which is available to authorized users.

## Background

Physical literacy is defined in this paper as the “motivation, confidence, physical competence, knowledge and understanding to value and take responsibility for engagement in physical activities for life” [1]. Interest in physical literacy has increased rapidly in recent years and programs, curricula, and policies intended to improve physical literacy are emerging, with some researchers and educators articulating that physical literacy is as important to develop as literacy and numeracy [2–4]. This is logical given the favourable associations between physically active lifestyles and a wide variety of health indicators [5]. Several countries have begun to incorporate the construct of physical literacy into their educational systems [1, 4, 6]; however, a global consensus on the definition of physical literacy is still lacking [7], and a recent systematic review concluded that little empirical research assessing physical literacy has been conducted to date [8]. Due to the limited amount of objective physical literacy data, the Canadian Assessment of Physical Literacy (CAPL) was developed.

The CAPL was developed and refined between 2009 and 2013, and its overall aim is to provide a reliable, feasible, and valid instrument to assess physical literacy in Canadian children [2, 9, 10]. It incorporates 25 measures within four interrelated domains: Physical Competence, Daily Behaviour, Knowledge and Understanding, and Motivation and Confidence. The CAPL scoring system was developed using a Delphi process with international experts in various fields representing the four domains [11]. An overall physical literacy score (out of 100) as well as individual domain scores are calculated using the CAPL. As advised by the Delphi expert panel, the Physical Competence and Daily Behaviour domains (each maximum 32 points) are weighted higher than the Knowledge and Understanding and the Motivation and Confidence domains (each maximum 18 points) due to the fact that the former are easier to assess objectively [9, 11].

Assessments and evaluation are very important in the education and health fields [12], with the assessment of physical fitness in North American children gaining prominence approximately 60 years ago [13]. For the past several decades there has been a contentious debate regarding the methods and appropriateness of physical fitness testing [14, 15]. The declining physical fitness levels in Canadian children [16] is concerning, as physical fitness has been found to be a powerful marker of health in children and adolescents [17]. Due to the controversy surrounding the assessment of physical fitness in children and youth, Lloyd et al. [12] suggested that instead of measuring only one component (i.e., physical fitness) in physical education classes, we should instead be assessing physical literacy, a broader, more holistic construct. As little is known regarding the physical literacy levels of Canadian children to date, and as monitoring and surveillance are required to assess interventions and trends, the aim of this paper was to establish the current physical literacy levels of Canadian children aged 8 to 12 years using the CAPL.

## Methods

### Study design

The Royal Bank of Canada Learn to Play – Canadian Assessment of Physical Literacy (RBC Learn to Play–CAPL) was a national, multi-site surveillance research project that investigated the physical literacy levels of Canadian children aged 8–12 years. This study collected cross-sectional data from 2014 to 2017 through convenience sampling methods, using study sites identified and selected through professional networks of the RBC Learn to Play–CAPL Principal Investigator (PI) while being attentive to geographic dispersion. The coordinating centre for the RBC Learn to Play–CAPL study was the Healthy Active Living and Obesity Research Group located at the Children’s Hospital of Eastern Ontario Research Institute (CHEO RI). The overall study protocol was initially approved by the CHEO Research Ethics Board. Each participating study site was subsequently required to obtain research ethics approval from their respective institution, as well as approval from local school boards and individual schools or organizations where data collection occurred. All children participating in the study provided verbal assent, and parents or legal guardians provided their written informed consent, before data collection began.

### Study sites

Eleven study sites (including the coordinating centre), geographically dispersed across seven Canadian provinces, were selected to participate in the RBC Learn to Play–CAPL study: Victoria, British Columbia; Calgary, Alberta; Lethbridge, Alberta; Winnipeg, Manitoba; Windsor, Ontario; North Bay, Ontario; Ottawa, Ontario (coordinating centre); Trois-Rivières, Québec; Halifax, Nova Scotia; Antigonish, Nova Scotia; and Charlottetown, Prince Edward Island. All study site PIs were brought in to the coordinating centre for a two-day standardized training workshop on the CAPL’s background, how the measures were to be administered, and how data were to be collected and entered; study site PIs were then able to practise the CAPL on a subset of participants not included in these analyses. Study site PIs then trained Research Assistants and undergraduate/graduate level students from their institutions as a data collection team. The goal was to collect data on 10,000 children distributed across participating sites.

### Participants and setting

Canadian children aged 8–12 years were recruited for the RBC Learn to Play–CAPL study. This specific age range was selected since the CAPL was initially designed for this age range and has undergone either validity and/or reliability testing only for children 8–12 years old [10]. Convenience sampling techniques (e.g., established contacts or relationships; schools that volunteered after being contacted by school boards; schools/summer camps in reasonable proximity) were used; children were predominantly recruited through elementary schools in urban, rural, and suburban locations, to ensure that children of different socioeconomic status and physical activity levels were reached. In order to meet the target sample size, sites were encouraged to approach municipal/community organizations, after-school care services, family programs, and summer camps. Elite sport teams were not targeted for data collection, as these groups were not representative of the population at large.

### CAPL scoring and measures

**Fig. 1:**
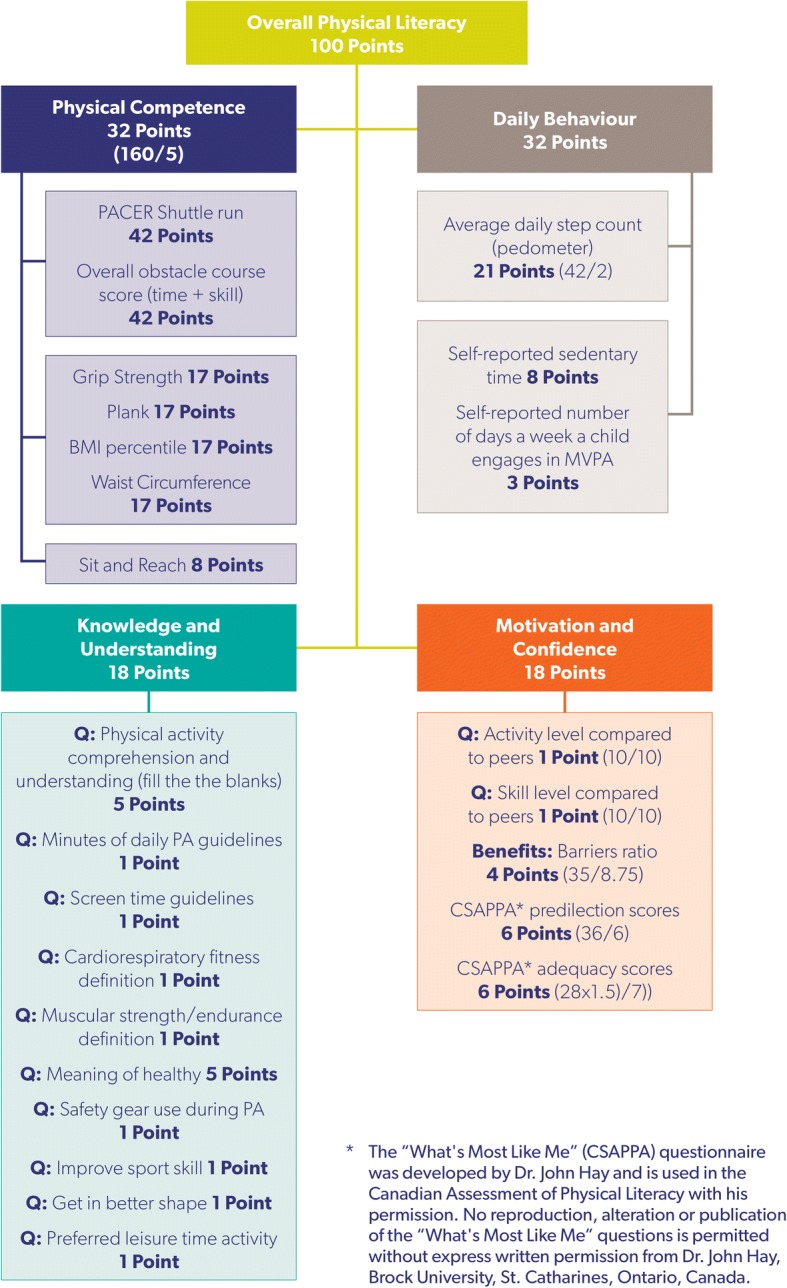
Canadian Assessment of Physical Literacy scoring system. BMI: body mass index; CSAPPA: Children’s Self-Perception of Adequacy in and Predilection for Physical Activity; MVPA: moderate- to vigorous-intensity physical activity; PA: physical activity; PACER: Progressive Aerobic Cardiovascular Endurance Run

The CAPL is comprised of domains and measures that align with the Canadian consensus and International Physical Literacy Association definitions of physical literacy [18]. The CAPL’s four domains are Daily Behaviour, Physical Competence, Knowledge and Understanding, and Motivation and Confidence. Daily Behaviour (32 points) and Physical Competence (32 points) were assigned more weight in the overall CAPL score (out of 100) than Knowledge and Understanding (18 points) and Motivation and Confidence (18 points), based on existing theoretical frameworks and input from the Delphi process (see Fig. 1) [11]. For each individual measure, domain score, and overall CAPL score, children are assigned one of four interpretation categories (stratified by age and gender) based on their performance: beginning, progressing, achieving, or excelling (see Additional file 1). The measures within the CAPL are standardized; whereby instructions, delivery, and scoring are uniform across testers and their respective study sites. Detailed descriptions of each measure are available online in the CAPL manual (https://www.capl-ecsfp.ca).

#### Daily behaviour

Children’s physical activity levels were both objectively measured and self-reported in the CAPL, as per input from experts participating in the tool’s development Delphi process [11]. Physical activity was objectively measured by an SC-StepRx pedometer (StepsCount, Deep River, ON, Canada). Children were instructed to wear the pedometer around their waist on the right hip for seven days (beginning the day after the pedometers were distributed by the research staff), and to complete a daily log sheet indicating the time the pedometer was put on in the morning, the time it was taken off at night, and if the pedometer was removed for any reason (e.g., swimming, bathing). For self-reported weekly physical activity levels, children were asked, “During the past week (seven days), on how many days were you physically active for a total of at least 60 minutes per day?” Response options ranged from 0 days to 7 days.

In addition to physical activity levels, children were asked to report their time spent in various sedentary behaviours. Participants were asked to self-report their time spent watching TV, playing video or computer games, using a computer for non-school work, and time spent sitting down doing non-screen-based activities outside of school time (e.g., reading a book, doing homework). Response options for each question were: “I did not spend time”, “Less than 1 hour”, “1 h”, “2 h”, “3 h”, “4 h”, and “5 or more hours”. Each question was asked for a typical school day and a typical weekend day. The sedentary behaviour questions, and the self-reported physical activity question, were based on the Youth Risk Behavior Surveillance System [19].

#### Physical competence

The Physical Competence domain assesses the musculoskeletal fitness, motor competence, and anthropometric characteristics of the child. Muscular strength was assessed by a Smedley III Analog Grip Strength Dynamometer (Creative Health Products, Ann Arbor, MI, USA). While standing, children were instructed to grasp the dynamometer with one hand and abduct that arm away from their torso (approximately 30–45 degrees). While keeping the elbow straight, children were told to “squeeze” the dynamometer handle by performing a fist motion. Children performed two trials with each hand, alternating between hands for each trial, and the maximum scores for each hand (kg) were combined to calculate the total score [16].

Trunk muscular endurance was measured using the isometric torso plank protocol [20]. Children were instructed to assume the plank position (i.e., push-up position but using their forearms for support instead of their palms) and maintain the isometric position for as long as they could without breaking form. Children were allowed one correction by the research staff to resume proper form. The measure concluded when the child either displayed volitional fatigue (e.g., dropping to their knees) or when a second break in form was observed. Only one trial was conducted and scores were recorded to the nearest 0.1 s.

Aerobic fitness was assessed using the Progressive Aerobic Cardiovascular Endurance Run (PACER) [21]. For the PACER, a progressive test, children and youth were asked to run back and forth between two parallel lines 20 m apart or 15 m apart (subsequently converted to 20-m distance score, according to Carrel et al. [22]). An audio recording paced the participants, beginning at a speed of 8.5 km/h and increasing by 0.5 km/h every consecutive minute. Participants continued until they were no longer able to keep pace with the audio recording for two consecutive laps, at which point their last completed lap was recorded.

Motor competence was evaluated using the Canadian Agility and Movement Skill Assessment (CAMSA), which is an obstacle-type course that combines both fundamental (jumping, sliding, catching, throwing, etc.) and complex (acceleration, deceleration, hand-eye coordination, etc.) movement skills [23]. Each child performed four trials on the CAMSA: two practice trials and two test trials. Children were scored on time (nearest 0.1 s) required to complete the CAMSA (range 1–14 points) and their ability to demonstrate the movement skill criteria (range 0–14 points) for a combined score out of 28. The best score out of the two test trials was used for CAPL scoring. The CAMSA has been shown to have good convergent validity (older age and boys achieved a higher total score); good inter-rater reliability evidence (intraclass correlation coefficient [ICC] = 0.99 for completion time and substantial for skill score ICC = 0.69); and moderate intra-rater reliability (ICC = 0.52) for skill score and excellent reliability for completion time (ICC = 0.99). Reliability was also excellent for completion time over a short (2–4 days; ICC = 0.84) or long (8–14 days; ICC = 0.82) interval, while skill score reliability was moderate (ICC = 0.46) over a short interval, and substantial (ICC = 0.74) over a long interval [24].

Static flexibility was assessed using the sit-and-reach protocol [16]. Children were instructed to sit on a floor mat with their legs fully extended in front of them, and the balls of their feet touching the Novel Acuflex I flexometer (Creative Health Products, Ann Arbor, MI, USA). While keeping their legs extended, children were advised to extend both arms toward their toes and stack their hands on top of one another. Children were then instructed to reach forward by performing trunk flexion and push the metal tracker on the flexometer as far as possible, holding the final end-point for five seconds. Children performed two trials, and the score from the best trial (nearest 0.5 cm) was used for CAPL scoring.

Height was measured in duplicate with a portable stadiometer (SECA, Hamburg, Germany) without footwear to the nearest 0.1 cm, and weight was measured with a digital scale (A&D Medical, Milpitas, CA, USA) or mechanical beam scale (if used weight was measured in duplicate) without footwear to the nearest 0.5 kg [16]. Body mass index (BMI) was calculated by dividing the child’s weight in kilograms by their height in metres squared, and converted to a BMI z-score using the World Health Organization (WHO)‘s BMI-for-age charts and formulae based on the LMS method [25]. Waist circumference was measured in duplicate using a non-elastic tape measure at the level of the iliac crest and recorded to the nearest 0.5 cm, with the average of the two measures used for analyses [16]. If duplicate measures varied by greater than 0.5 cm or 0.5 kg for the aforementioned protocols, a third measure was taken and an average of the closest two measures was recorded.

#### Knowledge and understanding

Children completed a 10-indicator Physical Literacy Knowledge Questionnaire, either in paper-and-pencil format or online through the CAPL website, to assess their knowledge and understanding of items related to physical activity. The questions were anchored in Canadian provincial curricula for physical and health education for children in grades 4 to 6 [26]. Children answered questions on a variety of topics, including knowledge of the Canadian Physical Activity Guidelines for Children and Youth [27], knowledge of the Canadian Sedentary Behaviour Guidelines for Children and Youth [28], knowledge of the terms “cardiorespiratory fitness” and “muscular strength”, knowledge of “what it means to be healthy” (matching the word “healthy” to various phrases), a comprehension and understanding paragraph (fill in the blanks with a word bank provided), knowledge of when to use safety equipment during activities (circling activities that are performed by the child and determining whether or not safety gear is needed for those activities), knowledge on how to improve sport skills and fitness, and responding to a question on their preferred leisure time activities (either active or inactive pursuits). The Physical Literacy Knowledge Questionnaire demonstrates good validity and feasibility in this age group and is available elsewhere in this supplement [26]. Knowledge scores increased with age (partial eta^2^ = 0.07) but were not related to gender, supporting the validity of the questionnaire. Test-retest reliability for the questionnaire score and individual questions was substantial to excellent for 71% of comparisons over a 2-day interval, but lower over a 7-day interval (53% substantial or excellent). More details on the Physical Literacy Knowledge Questionnaire are available in an accompanying manuscript [26].

#### Motivation and confidence

Children completed a five-indicator questionnaire, either in paper-and-pencil format or online through the CAPL website, to assess their motivation and confidence levels for physical activity. Children answered questions on a variety of motivation- and/or confidence-related constructs: a benefits-to-barriers ratio for physical activity was calculated from children rating their agreement on a scale of 1 to 5 (1 = disagree; 5 = agree) for 19 proposed items (10 barriers and nine benefits) [29]; adequacy and predilection subscales representing 17 items from the Children’s Self-Perceptions of Adequacy in, and Predilection for Physical Activity (CSAPPA) Scale were determined by children answering items using a structured alternative response format (scale of 1 to 4) [30]; and “activity levels compared to others” and “skill level compared to others” were determined by children completing one item for each construct using a 10-point scale (1 = “a lot less active” OR “others are better”; 10 = “a lot more active” OR “I’m a lot better”). The Adequacy and Predilection subscales have been shown to have good test-retest reliability and predictive validity [30].

### Paradata

Given the novel nature of this research, efforts were built into the larger study to better understand the consequences (e.g., refusals, adverse events) and inclusiveness of collecting physical literacy surveillance data. Accordingly, in a subset of 510 participants from six sites, we assessed refusal rates across the various measures. Among a subset of 1196 participants with detailed participation data, the prevalence and type of reported disabilities or medical conditions identified by parents was examined. Records of all 10,034 participants were checked for reporting of adverse events.

### Statistical analysis

Means and standard deviations were calculated by age and gender for all CAPL variables. Generalized additive models for location, scale, and shape (GAMLSS) were used to generate normative values for several variables in the RBC Learn to Play–CAPL dataset. GAMLSS models use different methods to treat over-dispersion, skewness, and kurtosis in a dependent variable within a univariate analysis as compared to more traditional regression models. The “gamlss” R package was used to fit the GAMLSS models [31]. Effect size differences between boys and girls were examined using the Cohen’s *d* method [32], reflecting the magnitude of the difference between groups. To examine differences across ages, unstandardized beta estimates from linear regression models regressing the variable of interest on age in years were divided by their standard deviation to provide the average effect size across age. Effect sizes were considered negligible if < 0.2, small if between 0.2 and 0.5, moderate if between 0.5 and 0.8, and important if > 0.8 [32]. All analyses were performed using R 3.5.0 (The R Foundation for Statistical Computing, Vienna, Austria).

## Results

A total of 10,034 children participated in the RBC Learn to Play–CAPL project. Table 1 shows the breakdown of the participants by gender and data collection site. Table 2 displays the RBC Learn to Play–CAPL descriptive statistics overall and stratified by gender. Overall, the mean age of the participants was 10.1 ± 1.2 years, with 50.1% (*n* = 5030) of the participants being girls.

**Table 1:** The number of study participants by gender and data collection site for the RBC Learn to Play–CAPL project

Site (city, province)	Number of schools/camps	Boys	Girls	Total
Antigonish, NS	11	515	549	1064
Calgary, AB	2	637	633	1270
Charlottetown, PEI	17	269	267	536
Halifax, NS	20	424	431	855
Lethbridge, AB	18	551	564	1115
North Bay, ON	20	533	589	1122
Ottawa, ON	43	430	448	878
Trois-Rivières, QC	3	67	48	115
Victoria, BC	6	268	231	499
Windsor, ON	29	670	608	1278
Winnipeg, MB	16	640	662	1302
Total	185	5004	5030	10034

*AB* Alberta, *BC* British Columbia, *CAPL* Canadian Assessment of Physical Literacy, *MB* Manitoba, *NS* Nova Scotia, *ON* Ontario, *PEI* Prince Edward Island, *RBC* Royal Bank of Canada, *QC* Québec

**Table 2:** RBC Learn to Play–CAPL descriptive statistics, overall and by gender

Variable	Overall		Boys		Girls		Boys vs. girls	
	n	Mean ± SD	n	Mean ± SD	n	Mean ± SD	Cohen’s *d*	95% CI
Age (years)	10,034	10.1 ± 1.2	5004	10.1 ± 1.2	5030	10.1 ± 1.2	−0.01	− 0.05, 0.03
Physical Competence score (/32)	9388	19.6 ± 4.4	4687	19.9 ± 4.7	4701	19.3 ± 4.1	0.14	0.10, 0.18
Sit-and-reach max score (cm)	9620	28.1 ± 8.4	4796	25.4 ± 7.6	4824	30.9 ± 8.3	**−0.69**	− 0.73, − 0.65
Total handgrip strength (kg)	9668	33.5 ± 9.4	4815	34.5 ± 9.6	4853	32.6 ± 9.2	0.21	0.17, 0.25
PACER (20 m laps)	9393	23.4 ± 14.1	4710	25.8 ± 15.8	4683	20.9 ± 11.6	0.35	0.31, 0.40
Plank time (sec)	9606	61.8 ± 43.8	4780	62.4 ± 44.8	4826	61.3 ± 42.9	0.02	−0.02, 0.06
Body mass index (kg/m^2^)	9455	19.0 ± 3.8	4716	18.9 ± 3.9	4739	19.0 ± 3.7	−0.01	−0.05, 0.03
Waist circumference (cm)	9395	67.3 ± 10.8	4677	67.4 ± 11.1	4718	67.2 ± 10.6	0.02	−0.02, 0.07
CAMSA max score (/28)	9488	20.6 ± 3.9	4752	21.0 ± 3.9	4736	20.3 ± 3.8	−0.17	−0.21, − 0.13
Daily Behaviour score (/32)	9783	18.6 ± 7.7	4867	18.6 ± 7.9	4916	18.5 ± 7.4	− 0.02	−0.06, 0.02
Daily steps taken	6640	11,512 ± 4006	3088	12,355 ± 4252	3552	10,779 ± 3624	0.40	0.35, 0.45
Physical activity guideline adherence (days/week)	9787	5.0 ± 1.9	4869	5.0 ± 2.0	4918	4.9 ± 1.9	−0.07	−0.11, − 0.04
Daily screen time (hrs)	9770	2.5 ± 1.9	4853	2.7 ± 2.1	4917	2.2 ± 1.8	− 0.30	−0.34, − 0.26
Daily non-screen sedentary time (hrs)	9776	1.7 ± 1.3	4863	1.6 ± 1.3	4913	1.7 ± 1.3	0.08	0.04, 0.12
Motivation and Confidence score (/18)	9625	12.5 ± 2.7	4778	12.7 ± 2.8	4847	12.2 ± 2.6	0.18	0.14, 0.22
Adequacy (/28)	9628	21.9 ± 4.3	4779	22.3 ± 4.3	4849	21.4 ± 4.2	−0.22	−0.26, − 0.18
Predilection (/36)	9628	28.8 ± 5.9	4779	29.0 ± 6.0	4849	28.7 ± 5.8	− 0.05	−0.09, − 0.01
Benefits-to-barriers ratio (/4)	9746	1.6 ± 1.2	4855	1.6 ± 1.2	4891	1.5 ± 1.1	− 0.12	−0.16, − 0.08
Physical activity level compared to peers (/10)	9865	7.2 ± 2.1	4906	7.3 ± 2.2	4959	7.0 ± 2.0	0.12	0.08, 0.16
Skill level compared to peers (/10)	9863	6.7 ± 2.3	4906	7 ± 2.4	4957	6.4 ± 2.2	0.27	0.23, 0.31
Knowledge and Understanding score (/18)	9797	12.0 ± 2.7	4870	11.8 ± 2.8	4927	12.2 ± 2.6	0.11	0.07, 0.15
Minutes of daily MVPA (/1)	9833	0.6 ± 0.5	4892	0.7 ± 0.5	4941	0.6 ± 0.5	0.15	0.11, 0.19
Minutes of daily screen time (/1)	9837	0.2 ± 0.4	4893	0.2 ± 0.4	4944	0.1 ± 0.3	−0.17	−0.21, −0.13
Cardiorespiratory fitness definition (/1)	9822	0.6 ± 0.5	4882	0.5 ± 0.5	4940	0.6 ± 0.5	0.11	0.07, 0.15
Muscular endurance definition (/1)	9829	0.7 ± 0.4	4890	0.7 ± 0.4	4939	0.8 ± 0.4	0.10	0.06, 0.13
Healthy definition (/5)	9856	4.0 ± 0.9	4908	3.9 ± 1.0	4948	4.0 ± 0.9	−0.18	−0.22, − 0.14
Fill in the missing words (/5)	9814	3.6 ± 1.5	4880	3.6 ± 1.5	4934	3.6 ± 1.4	0.02	−0.02, 0.06
Safety gear during physical activity (/1)	9856	0.3 ± 0.3	4908	0.3 ± 0.3	4948	0.4 ± 0.3	−0.35	−0.39, − 0.31
How to get better at a sport skill (/1)	9798	0.5 ± 0.5	4871	0.5 ± 0.5	4927	0.5 ± 0.5	0.03	−0.01, 0.07
How to improve physical fitness (/1)	9804	0.8 ± 0.4	4872	0.8 ± 0.4	4932	0.8 ± 0.4	0.08	0.04, 0.12
Preferred leisure-time activity (/1)	9835	0.7 ± 0.4	4894	0.7 ± 0.5	4941	0.8 ± 0.4	−0.14	−0.17, − 0.10
Physical literacy score (/100)	9781	62.7 ± 12.2	4866	63.1 ± 13.0	4915	62.2 ± 11.3	− 0.07	−0.11, − 0.03

*CAMSA* Canadian Agility and Movement Skill Assessment, *CAPL* Canadian Assessment of Physical Literacy, *CI* confidence interval, *MVPA* moderate- to vigorous-intensity physical activity, *PACER* Progressive Aerobic Cardiovascular Endurance Run, *RBC* Royal Bank of Canada, *SD* standard deviation

Important (> 0.8) effect sizes are in bold

**Fig. 2:**
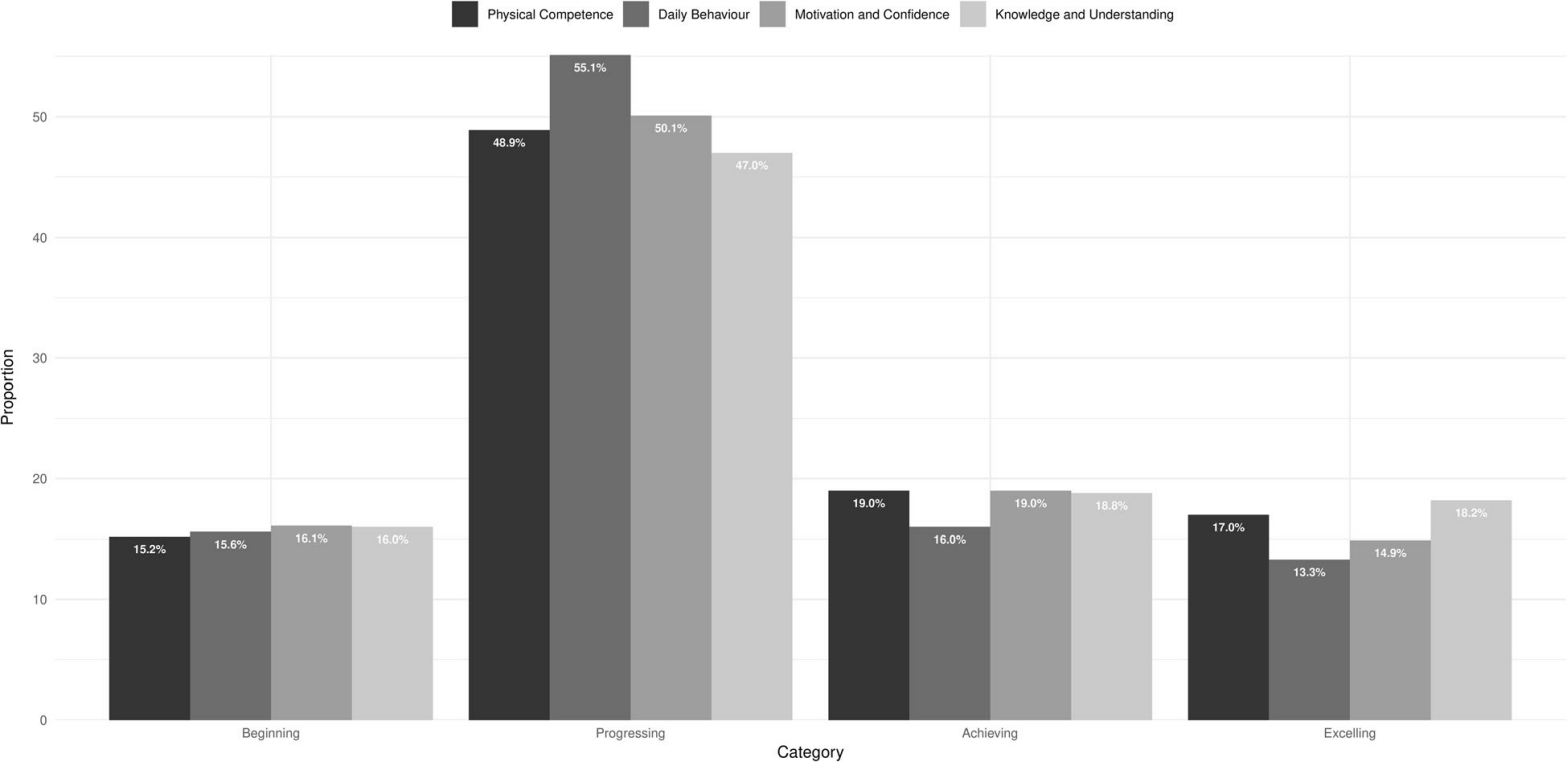
Proportion of participants in each of the four interpretation categories by domain

Total physical literacy scores (out of 100) were on average 63.1 ± 13.0 for boys and 62.2 ± 11.3 for girls. For boys and girls respectively, domain scores were: 19.9 ± 4.7 and 19.3 ± 4.1 for Physical Competence (out of 32 points); 18.6 ± 7.9 and 18.5 ± 7.4 for Daily Behaviour (out of 32 points); 12.7 ± 2.8 and 12.2 ± 2.6 for Motivation and Confidence (out of 18 points); and 11.8 ± 2.8 and 12.2 ± 2.6 for Knowledge and Understanding (out of 18 points). For the total physical literacy score and the domain scores there were negligible differences observed between genders (Cohen’s *d* range: 0.02 to 0.18). Using the CAPL’s interpretation system (which divides the participants into four categories: beginning, progressing, achieving, and excelling), based on the average total physical literacy score, both boys and girls would be classified as progressing. The individual domain scores for both genders would be classified as progressing for the Physical Competence, Daily Behaviour, and Motivation and Confidence domains, whereas they would be classified as achieving in the Knowledge and Understanding domain. The proportion of participants in each of the four interpretation categories by domain are presented in Fig. 2.

Table 3 shows the RBC Learn to Play–CAPL overall descriptive statistics stratified by age (in one-year increments). The total physical literacy score, as well as the domain scores for Physical Competence and for Knowledge and Understanding, increased with age. The Daily Behaviour domain score decreased with age (effect size negligible; Daily Behaviour domain score decreased by 0.65 units on average as age increased by one year), whereas the Motivation and Confidence domain score exhibited no age-related differences.

**Table 3:** RBC Learn to Play–CAPL descriptive statistics, by age

Variable	8 years		9 years		10 years		11 years		12 years	
	n	Mean ± SD	n	Mean ± SD	n	Mean ± SD	n	Mean ± SD	n	Mean ± SD
Age (years)	1117	8.5 ± 0.3	1958	9.5 ± 0.3	2488	10.5 ± 0.3	3222	11.5 ± 0.3	1144	12.3 ± 0.2
Physical Competence score (/32)	1068	18.4 ± 4.0	1851	19.0 ± 4.3	2343	19.3 ± 4.3	3033	20.2 ± 4.4	1053	21.0 ± 4.5
Sit-and-reach max score (cm)	1088	29.1 ± 7.3	1878	28.7 ± 8.0	2379	28.0 ± 8.2	3096	27.8 ± 8.7	1082	28.0 ± 9.2
Total handgrip strength (kg)	1087	26.2 ± 6.0	1875	29.5 ± 7.0	2414	32.3 ± 8.0	3108	36.6 ± 9.1	1088	41.4 ± 10.6
PACER (20 m laps)	1045	20.7 ± 13.0	1833	21.8 ± 12.9	2341	22.4 ± 13.3	3032	24.7 ± 14.6	1047	27.1 ± 16.1
Plank time (sec)	1080	54.7 ± 39.5	1869	61.3 ± 45.5	2391	60.5 ± 42.7	3093	64.3 ± 45.0	1077	65.1 ± 42.8
Body mass index (kg/m^2^)	1064	17.3 ± 2.8	1860	18.3 ± 3.5	2378	18.9 ± 3.7	3051	19.6 ± 3.9	1058	20.0 ± 4.1
Waist circumference (cm)	1075	61.3 ± 7.9	1837	64.4 ± 9.6	2349	67.2 ± 10.4	3036	69.8 ± 11.1	1054	71.3 ± 11.0
CAMSA max score (/28)	1076	18 ± 4.1	1846	19.7 ± 3.8	2357	20.5 ± 3.7	3068	21.7 ± 3.5	1046	22.3 ± 3.5
Daily Behaviour score (/32)	1089	19.6 ± 7.4	1911	19.7 ± 7.4	2461	18.6 ± 7.5	3206	17.7 ± 7.7	1112	17.7 ± 8.2
Daily steps taken	706	12,160 ± 3923	1268	12,094 ± 4019	1727	11,578 ± 4069	2163	11,078 ± 3919	728	10,920 ± 3972
Physical activity guideline adherence (days/week)	1092	4.8 ± 2.2	1913	5.0 ± 2.0	2461	5.0 ± 1.9	3205	4.9 ± 1.9	1112	4.9 ± 1.8
Daily screen time (hrs)	1086	2.2 ± 2.0	1905	2.3 ± 2.0	2459	2.4 ± 1.9	3204	2.6 ± 1.9	1112	2.7 ± 2.0
Daily non-screen sedentary time (hrs)	1080	1.4 ± 1.3	1914	1.5 ± 1.3	2458	1.6 ± 1.3	3206	1.8 ± 1.3	1114	1.9 ± 1.3
Motivation and Confidence score (/18)	1058	12.3 ± 2.5	1873	12.6 ± 2.5	2405	12.6 ± 2.7	3161	12.4 ± 2.8	1124	12.5 ± 3.0
Adequacy (/28)	1061	21.7 ± 4.0	1877	21.9 ± 4.1	2403	22.0 ± 4.3	3159	21.7 ± 4.4	1123	22.1 ± 4.5
Predilection (/36)	1061	28.5 ± 5.7	1877	29.0 ± 5.7	2403	29.1 ± 5.9	3159	28.6 ± 6.0	1123	28.7 ± 6.2
Benefits-to-barriers ratio (/4)	1073	1.5 ± 1.3	1900	1.6 ± 1.2	2450	1.6 ± 1.2	3193	1.6 ± 1.1	1127	1.6 ± 1.2
Physical activity level compared to peers (/10)	1102	7.5 ± 2.2	1926	7.3 ± 2.1	2474	7.2 ± 2.1	3217	7.0 ± 2.0	1142	7.1 ± 2.1
Skill level compared to peers (/10)	1102	7.0 ± 2.5	1925	6.9 ± 2.4	2475	6.7 ± 2.3	3216	6.5 ± 2.2	1141	6.7 ± 2.2
Knowledge and Understanding score (/18)	1092	10.4 ± 2.7	1916	11.2 ± 2.6	2461	12.1 ± 2.7	3210	12.7 ± 2.6	1115	12.9 ± 2.5
Minutes of daily MVPA (/1)	1099	0.5 ± 0.5	1927	0.5 ± 0.5	2471	0.6 ± 0.5	3216	0.7 ± 0.5	1117	0.7 ± 0.4
Minutes of daily screen time (/1)	1103	0.1 ± 0.3	1927	0.1 ± 0.3	2468	0.2 ± 0.4	3217	0.2 ± 0.4	1118	0.2 ± 0.4
Cardiorespiratory fitness definition (/1)	1101	0.4 ± 0.5	1919	0.5 ± 0.5	2466	0.6 ± 0.5	3214	0.7 ± 0.5	1118	0.7 ± 0.5
Muscular endurance definition (/1)	1102	0.6 ± 0.5	1925	0.7 ± 0.5	2467	0.8 ± 0.4	3213	0.8 ± 0.4	1118	0.8 ± 0.4
Healthy definition (/5)	1107	3.9 ± 1.0	1933	3.9 ± 0.9	2476	4.0 ± 0.9	3217	4.0 ± 1.0	1118	4.0 ± 0.9
Fill in the missing words (/5)	1098	2.8 ± 1.5	1921	3.2 ± 1.4	2468	3.6 ± 1.4	3208	3.9 ± 1.4	1116	4.0 ± 1.3
Safety gear during physical activity (/1)	1107	0.3 ± 0.3	1933	0.3 ± 0.3	2476	0.3 ± 0.3	3217	0.3 ± 0.3	1118	0.3 ± 0.3
How to get better at a sport skill (/1)	1092	0.4 ± 0.5	1918	0.5 ± 0.5	2460	0.5 ± 0.5	3210	0.5 ± 0.5	1114	0.6 ± 0.5
How to improve physical fitness (/1)	1092	0.7 ± 0.5	1921	0.8 ± 0.4	2462	0.8 ± 0.4	3211	0.8 ± 0.4	1114	0.8 ± 0.4
Preferred leisure-time activity (/1)	1098	0.6 ± 0.5	1928	0.7 ± 0.5	2473	0.7 ± 0.4	3214	0.7 ± 0.4	1118	0.7 ± 0.4
Physical literacy score (/100)	1093	60.9 ± 11.2	1915	62.4 ± 11.5	2456	62.6 ± 12.2	3202	62.9 ± 12.5	1112	64.2 ± 13.5

*CAMSA* Canadian Agility and Movement Skill Assessment, *CAPL* Canadian Assessment of Physical Literacy, *MVPA* moderate- to vigorous-intensity physical activity, *PACER* Progressive Aerobic Cardiovascular Endurance Run, *RBC* Royal Bank of Canada, *SD* standard deviation

Absolute scores on Physical Competence measures were on average 28.1 ± 8.4 cm for sit-and-reach flexibility, 33.5 ± 9.4 kg for handgrip strength (right + left), 23.4 ± 14.1 laps for the PACER shuttle run, 61.8 ± 43.8 s for the isometric plank, 19.0 ± 3.8 kg/m^2^ for BMI, 67.3 ± 10.8 cm for waist circumference, and 20.6 ± 3.9 out of 28 points for the CAMSA. Scores for handgrip strength, PACER, plank, and CAMSA were higher in boys than girls, and in older children than in younger children. Girls and younger children had better scores on sit-and-reach flexibility compared to boys and older children, respectively. The largest differences between boys and girls in the Physical Competence domain were for sit and reach (25.4 ± 7.6 vs. 30.9 ± 8.3 cm, respectively; Cohen’s *d* = 0.69), the PACER (25.8 ± 15.8 vs. 20.9 ± 11.6 laps, respectively; Cohen’s *d* = 0.35), and handgrip strength (34.5 ± 9.6 vs. 32.6 ± 9.2 kg, respectively; Cohen’s *d* = 0.21). The rest of the effect sizes between genders for the Physical Competence domain measures were considered negligible (Cohen’s *d* range: 0.01 to 0.17).

For the Daily Behaviour domain, daily step counts were higher in boys than in girls (12,355 ± 4252 vs. 10,779 ± 3624 steps; Cohen’s *d* = 0.40) and decreased with age. Self-reported physical activity (i.e., number of days adhering to 60 min of moderate- to vigorous-intensity physical activity [MVPA] per day) was similar across genders and ages (approximately 5 out of 7 days per week). Boys reported more screen time than girls (2.7 ± 2.1 vs. 2.2 ± 1.8 h; Cohen’s *d* = 0.30), with screen time increasing with age.

For the measures in the Motivation and Confidence domain, the effect sizes between genders were considered negligible to small (Cohen’s *d* range: 0.05 to 0.22) and no age-related differences were observed. With regard to the measures in the Knowledge and Understanding domain, all of the effect sizes between boys and girls were considered negligible (Cohen’s *d* range: 0.02 to 0.18), except for the question pertaining to safety gear while being physically active, where there was a small effect (Cohen’s *d* = 0.35), with girls outperforming boys. Overall, there was a pattern showing that the measures within the Knowledge and Understanding domain increased with age (effect size estimates range from 0.03 to 0.21) (see Additional file 2).

Additional file 2 provides the overall descriptive statistics stratified by age and gender. The percentiles for the total physical literacy score, domain scores, the individual components in the Physical Competence domain, and daily step counts are provided in Additional file 3.

Paradata from the RBC Learn to Play–CAPL project found that in a subset of 510 participants from six sites with detailed information about the protocol completion, 5% or less of the participants refused to complete one or more CAPL protocols. Among the CAPL protocols, rates of refusal for the PACER, waist circumference, pedometer step, and weight protocols were similar, ranging from 3.7 to 5.4%. Refusals occurred among 2 to 3% of participants for the plank, sit and reach, height, handgrip, and CAMSA protocols. Only three children (0.05%) among this subset refused to complete the questionnaire protocols. A detailed analysis of missing data from the RBC Learn to Play–CAPL project is provided in the paper by Delisle Nyström et al. [33].

Although participants were not required to disclose personal information related to disabilities or medical conditions, parents or legal guardians were asked to indicate if a doctor had said that there were some types of physical activity that their child should not perform. Among a subset of 1196 (*n* = 586, 49% boys, mean age 10.1 years) participants with detailed participation data, 104 children (9%) had disabilities or medical conditions identified by their parents. The disabilities/medical conditions reported included asthma/breathing conditions (*n* = 50, 4%), developmental disability (*n* = 12, 1%), physical or vision disability (*n* = 10, 1%), learning disability/attention deficit hyperactivity disorder (*n* = 8, 1%), concussion/bleeding disorder (n = 8, 1%), heart condition (*n* = 4, 0.3%), arthritis (*n* = 2, 0.2%), migraine (n = 2, 0.2%), epilepsy/seizure risk (n = 2, 0.2%), and other medical conditions (*n* = 6, 0.5%).

All study sites were required to immediately report adverse events (e.g., injuries or illness) to the study coordinating centre. Of the 10,034 participants in the RBC Learn to Play–CAPL project, there were only two reported adverse events. One child twisted their ankle when reversing direction in the PACER shuttle run, and another child had a similar injury when they stepped on a hoop during the CAMSA assessment. Both incidents were minor injuries.

## Discussion

This is the first study to report the physical literacy levels of a large sample of Canadian children. Overall, there were no large differences between boys’ and girls’ total physical literacy scores or the individual domain scores. Using the CAPL’s interpretation system [34], the results show that on average the total physical literacy score, as well as the Physical Competence, Daily Behaviour, and Motivation and Confidence domains are at the “progressing” level, and only the Knowledge and Understanding domain is at the desired “achieving” level. These overall “low” scores could be due to societal change where, from a young age, children’s free time is more focused on screens than active play. The reduced active play time could influence the scores for overall physical literacy as well as for the Physical Competence, Daily Behaviour, and Motivation and Confidence domains, as children are not developing the skills needed to adequately achieve in these areas. It is important to note that the interpretation of the total score and the domain scores is based upon cut-points informed by criterion thresholds for measures where such thresholds exist (e.g., step counts, BMI), and upon normative thresholds believed to be consistent with current trends in obesity, fitness, and physical inactivity when criterion thresholds were not available. Future research needs to further validate these thresholds. However, the average values and the overall classification of “progressing” for the total physical literacy score and three of the four domain scores demonstrates that there is room for improvement in Canadian children’s physical literacy, and that greater efforts for the promotion of physical literacy are needed.

### Physical competence

For the individual measures within the Physical Competence domain, we found boys had higher scores than girls for handgrip strength, PACER, plank, and CAMSA; whereas girls scored higher on the sit-and-reach measure. For handgrip strength, a previous Canadian study also found that boys scored higher on handgrip strength than girls [16]. However, the children in the RBC Learn to Play–CAPL project had higher mean values than the children from the Canadian Health Measures Survey (CHMS) 2007–2009 [16] (boys: 34.5 kg vs. 25 kg, respectively; and girls: 32.6 kg vs. 23 kg, respectively). The difference in the mean values is probably due to the age differences in the two studies (8- to 12-year-olds in the RBC Learn to Play–CAPL study and 6- to 10-year-olds in the CHMS). Using the sex- and age-specific cut-points created by Tomkinson et al. [35] for the number of completed laps in the 20-m shuttle run, boys aged 9–12 years from the RBC Learn to Play–CAPL study would be categorized in the 30th percentile; whereas girls would be classified between the 20th and < 40th percentiles. Using the quintile framework, this would classify both boys and girls of all age categories as having low cardiorespiratory endurance [35]. For sit-and-reach flexibility, the CHMS 2007–2009 [16] also found that girls had higher scores than boys, with similar results being observed between both studies. To date, there are no comparative data for the plank or CAMSA; therefore, the RBC Learn to Play–CAPL project is providing baseline measures for these important indicators of physical competence from a large sample of Canadian children.

Using the RBC Learn to Play–CAPL data, Lang et al. [36] found positive relationships between physical literacy and cardiorespiratory fitness. Furthermore, a positive reciprocal relationship between physical activity and motor competence has been demonstrated across children and youth aged 6 to 13 years, with further evidence of a mediating effect of aerobic fitness (VO_2_ peak) in both directions [37]. These findings support the development and promotion of interventions that target each of these domains of physical literacy to activate positive feedback loops amongst the domains and to facilitate physical literacy development.

The two anthropometric measurements included in the Physical Competence domain were BMI and waist circumference, with negligible differences being observed between genders for both indicators. Using the WHO’s BMI-for-age percentiles [25], which are specific for sex and age, we found that boys and girls of all ages were within or close to being within the 75th and 85th percentile, which would classify the children on the higher end of normal weight on average. With regard to waist circumference, using the Centers for Disease Control and Prevention age- and sex-specific reference values [38], boys and girls would be classified on average as normal. Boys aged 8–12 years were between the 50th and 75th percentile, with 11-year-olds being just below the 50th percentile. For girls, 8-year-olds were between the 50th and 75th percentile whereas girls aged 9–12 years were between the 25th and 50th percentiles.

### Daily behaviour

The individual measures within the Daily Behaviour domain showed a small difference between boys and girls for average daily step counts and daily screen time, with boys accumulating more steps and more screen time than girls. Furthermore, it was observed that daily step counts decreased with age in both boys and girls. Our findings agree with those from the CHMS (compiled data from 2007 to 2009, 2009–2011, 2012–2013, and 2014–2015 cycles), where they also found similar patterns using objectively measured physical activity data [39]. The average step counts for boys and girls in the RBC Learn to Play–CAPL project was 12,355 and 10,779 steps, respectively, with only 27% of boys and 14% of girls meeting the 12,000 recommended steps per day, which is equivalent to 60 min of MVPA [40, 41]. The proportion meeting the 12,000 step recommendation is slightly lower than that from the Physical Activity Levels Among Youth study (41% of 5- to 19-year-olds; 2014–2016 data) from the Canadian Fitness and Lifestyle Research Institute [41]. Further, the average values obtained in this study are slightly lower than those obtained in the 2007–2009 CHMS, where boys and girls had on average 13,217 and 11,745 steps, respectively [42]. As stated previously, the observed differences are possibly due to the age differences between the two studies: 8- to 12-year-olds in RBC Learn to Play–CAPL and 6- to 10-year-olds in CHMS.

Boys and girls participating in the RBC Learn to Play–CAPL project reported being engaged in screen-based activities for on average 2.7 and 2.2 h/day, respectively, which exceeds the recommendation of ≤2 h of recreational screen time daily [43]. In the 2009–2011 and 2012–2013 CHMS [44], boys and girls aged 5 to 11 years reported (with parental assistance) a similar amount of screen time (2.4 and 2.3 h/day, respectively) compared to the participants in the present study. Using the information from RBC Learn to Play–CAPL participants who had provided complete and valid pedometer data, Belanger et al. [45] found that 20% of children met the physical activity guidelines and 57% met screen time recommendations. Given the consistently low daily levels of physical activity and excess screen time found here and reported in other studies, it is clear that more health promotion and policy work needs to be done to improve the Daily Behaviour domain of children’s physical literacy.

### Knowledge and understanding

The physical literacy knowledge questionnaire was based on existing physical and health education curricula, and specifically developed for use within the RBC Learn to Play–CAPL project; therefore, no comparative data exist. Longmuir et al. [26] investigated the feasibility, validity, and reliability of the questionnaire within a subset of the RBC Learn to Play–CAPL participants and concluded that it was a feasible, valid, and reliable tool to assess knowledge in 8- to 12-year-old Canadian children. Overall, the Knowledge and Understanding domain score did not differ by gender; however, an increase in the domain score was observed with age, as might be expected. Numerous governments and public health agencies have been working on increasing the public’s knowledge regarding the amount of physical activity that is needed for health benefits, with the hope that greater knowledge will lead to better decisions regarding physical activity. Very few studies have been conducted to date investigating children’s knowledge of physical activity guidelines and their physical activity levels. A study by Best et al. [46] found that knowledge of the physical activity guidelines was not an important predictor of physical activity in children and youth aged 11 to 16 years. However, another study by Xu et al. [47] found that Chinese children in grades 4 through 7 who became more aware of the relationships between obesity and physical activity significantly increased the frequency and amount of time spent on physical activity. Given the mixed evidence regarding the associations between knowledge of physical activity guidelines and actual physical activity levels, this area warrants further investigation.

### Motivation and confidence

Motivation is an important predictor and self-efficacy an important correlate of physical activity in children and youth [48, 49]. Within the Motivation and Confidence domain, negligible to small effect sizes were observed in the RBC Learn to Play–CAPL dataset for the individual measures between genders, and no age-related differences were observed. Participants’ perceived levels of adequacy and predilection for physical activity were moderately related to cardiorespiratory fitness [50], which has also been observed in another study [51]. These findings lend support to the importance of considering psychological factors when creating physical literacy interventions.

### Strengths and limitations

The CAPL is a large field-based assessment battery that includes 25 measures within four domains. A total of 10,034 children participated in the RBC Learn to Play–CAPL project; however, due to the large number of assessments, there were a lot of missing data. Delisle Nyström et al. [33] conducted an exploratory analysis of these missing data, and found that the pedometer step counts accounted for the greatest source of missing data (33.8%), followed by the components of the Physical Competence domain (3.6–6.4%), and the CSAPPA subscales (4.0%). To reduce the burden of the CAPL battery of tests, Gunnell et al. [52] conducted factor analyses to create a shorter and more theoretically aligned CAPL version. Through this work it was found that CAPL could be reduced to 14 indicators across the same four domains, and the revised version is now referred to as CAPL-2 [53].

This is the first study to provide descriptive and normative percentile data for physical literacy from a large sample of Canadian children, providing a baseline to be used for future comparisons, informing policy, and assessing interventions. The findings are strengthened by the large and diverse sample size, the reliable and valid protocols that were used to assess physical literacy [20, 24, 26], the standardized methods used in data collection, and the ability of the CAPL to assess physical literacy without bias across children aged 8 to 12 years [54] and in varying weight classes [55]. A limitation of the study was the use of convenience sampling and therefore the findings may not be generalizable to all Canadian children aged 8 to 12 years. However, all sites were instructed to collect data in locations that offered varied levels of urbanization (i.e., rural, suburban, and urban) and socioeconomic status. Although not assessed, diversity is likely since most data were collected within schools, which reach a broad spectrum of children from across social classes and ethnicities. Nevertheless, information on socioeconomic status and cultural background was not collected, and this information may be important to interpretation of the findings. Future research should consider whether a maturation measure in the CAPL may provide additional insights on differences observed between genders and ages, especially when focusing on older children or adolescents. Despite these limitations, the characteristics of the RBC Learn to Play–CAPL sample were similar to nationally representative CHMS data (e.g., prevalence of overweight and obesity 36.4% [55] and 31.4% [56], respectively).

## Conclusions

These results provide the largest and most comprehensive assessment of the physical literacy of Canadian children to date, providing a “state of the nation” baseline. They can be used to monitor and inform domestic changes in this area of study going forward. Importantly, they highlight the need to enhance efforts to promote the physical literacy of Canadian children.

## Additional files

Additional file 1: CAPL interpretation categories for each domain and each measure by age and gender. (DOCX 112 kb)

Additional file 2: RBC Learn to Play–CAPL descriptive statistics stratified by gender and age. (DOCX 50 kb)

Additional file 3: Percentiles for the total physical literacy score, domain scores, the individual components in the physical competence domain, and daily step counts. (DOCX 132 kb)

## Abbreviations

BMI: Body mass index
CAMSA: Canadian Agility and Movement Skill Assessment;
CAPL: Canadian Assessment of Physical Literacy
CHEO RI: Children’s Hospital of Eastern Ontario Research Institute
CHMS: Canadian Health Measures Survey
CSAPPA: Children’s Self-Perceptions of Adequacy in, and Predilection for Physical Activity
GAMLSS: Generalized additive models for location, scale, and shape
ICC: Intraclass correlation coefficient
MVPA: Moderate- to vigorous-intensity physical activity
PACER: Progressive Aerobic Cardiovascular Endurance Run
PI: Principal investigator
RBC: Royal Bank of Canada
WHO: World Health Organization
