# Supplementary material for: Physical literacy levels of Canadian children aged 8–12 years: descriptive and normative results from the RBC Learn to Play–CAPL project
Source: BMC Public Health. 2018 Oct 2;18(Suppl 2):1036. doi: 10.1186/s12889-018-5891-x (PMC6167776; doi:10.1186/s12889-018-5891-x)
Supplement: Supplementary file 1 — CAPL interpretation categories for each domain and each measure by age and gender. (DOCX 112 kb) [file 12889_2018_5891_MOESM1_ESM.docx]

| **GAMLSS (Box-Cox Power Exponential)** | | | | | | | | | | | | |
| --- | --- | --- | --- | --- | --- | --- | --- | --- | --- | --- | --- | --- |
| **Sit and Reach (cm)** | | **Beginning** | | **Progressing** | | **Progressing** | | **Achieving** | | **Achieving** | | **Excelling** |
| **Girls** | | **Less than** | | **Lower** | | **Upper** | | **Lower** | | **Upper** | | **Above** |
| 8 years | | 22.4 | | 22.4 | | 33.7 | | 33.8 | | 38.7 | | 38.7 |
| 9 years | | 22.5 | | 22.5 | | 33.9 | | 34.0 | | 39.0 | | 39.0 |
| 10 years | | 22.7 | | 22.7 | | 34.1 | | 34.2 | | 39.2 | | 39.2 |
| 11 years | | 22.9 | | 22.9 | | 34.4 | | 34.5 | | 39.6 | | 39.6 |
| 12 years | | 23.3 | | 23.3 | | 35.0 | | 35.1 | | 40.3 | | 40.3 |
| **GAMLSS (Normal)** | | | | | | | | | | | | |
| **Sit and Reach (cm)** | **Beginning** | | **Progressing** | | **Progressing** | | **Achieving** | | **Achieving** | | **Excelling** | |
| **Boys** | **Less than** | | **Lower** | | **Upper** | | **Lower** | | **Upper** | | **Above** | |
| 8 years | 20.4 | | 20.4 | | 30.4 | | 30.5 | | 35.2 | | 35.2 | |
| 9 years | 19.7 | | 19.7 | | 29.6 | | 29.7 | | 34.4 | | 34.4 | |
| 10 years | 18.9 | | 18.9 | | 28.8 | | 28.9 | | 33.6 | | 33.6 | |
| 11 years | 18.1 | | 18.1 | | 28.0 | | 28.1 | | 32.9 | | 32.9 | |
| 12 years | 17.3 | | 17.3 | | 27.2 | | 27.3 | | 32.1 | | 32.1 | |

| **GAMLSS (Box-Cox Power Exponential)** | | | | | | | | | | | | |
| --- | --- | --- | --- | --- | --- | --- | --- | --- | --- | --- | --- | --- |
| **Handgrip (kg)** | | **Beginning** | | **Progressing** | | **Progressing** | | **Achieving** | | **Achieving** | | **Excelling** |
| **Girls** | | **Less than** | | **Lower** | | **Upper** | | **Lower** | | **Upper** | | **Above** |
| 8 years | | 18.9 | | 18.9 | | 26.3 | | 26.4 | | 30.5 | | 30.5 |
| 9 years | | 20.9 | | 20.9 | | 29.0 | | 29.1 | | 33.5 | | 33.5 |
| 10 years | | 23.0 | | 23.0 | | 31.9 | | 32.0 | | 37.0 | | 37.0 |
| 11 years | | 25.8 | | 25.8 | | 35.9 | | 36.0 | | 41.6 | | 41.6 |
| 12 years | | 29.7 | | 29.7 | | 41.3 | | 41.4 | | 47.8 | | 47.8 |
| **GAMLSS (Box-Cox Power Exponential)** | | | | | | | | | | | | |
| **Handgrip (kg)** | **Beginning** | | **Progressing** | | **Progressing** | | **Achieving** | | **Achieving** | | **Excelling** | |
| **Boys** | **Less than** | | **Lower** | | **Upper** | | **Lower** | | **Upper** | | **Above** | |
| 8 years | 19.9 | | 19.9 | | 27.7 | | 27.8 | | 32.1 | | 32.1 | |
| 9 years | 22.5 | | 22.5 | | 31.3 | | 31.4 | | 36.3 | | 36.3 | |
| 10 years | 24.8 | | 24.8 | | 34.6 | | 34.7 | | 40.0 | | 40.0 | |
| 11 years | 27.1 | | 27.1 | | 37.8 | | 37.9 | | 43.8 | | 43.8 | |
| 12 years | 31.2 | | 31.2 | | 43.5 | | 43.6 | | 50.4 | | 50.4 | |

| **GAMLSS (Box-Cox Power Exponential)** | | | | | | | | | | | | |
| --- | --- | --- | --- | --- | --- | --- | --- | --- | --- | --- | --- | --- |
| **PACER (20-metre laps)** | | **Beginning** | | **Progressing** | | **Progressing** | | **Achieving** | | **Achieving** | | **Excelling** |
| **Girls** | | **Less than** | | **Lower** | | **Upper** | | **Lower** | | **Upper** | | **Above** |
| 8 years | | 9 | | 9 | | 19 | | 20 | | 27 | | 27 |
| 9 years | | 10 | | 10 | | 21 | | 22 | | 29 | | 29 |
| 10 years | | 10 | | 10 | | 21 | | 22 | | 30 | | 30 |
| 11 years | | 11 | | 11 | | 23 | | 24 | | 32 | | 32 |
| 12 years | | 12 | | 12 | | 26 | | 27 | | 36 | | 36 |
| **GAMLSS (Box-Cox Power Exponential)** | | | | | | | | | | | | |
| **PACER (20-metre laps)** | **Beginning** | | **Progressing** | | **Progressing** | | **Achieving** | | **Achieving** | | **Excelling** | |
| **Boys** | **Less than** | | **Lower** | | **Upper** | | **Lower** | | **Upper** | | **Above** | |
| 8 years | 10 | | 10 | | 25 | | 26 | | 37 | | 37 | |
| 9 years | 10 | | 10 | | 27 | | 28 | | 39 | | 39 | |
| 10 years | 11 | | 11 | | 28 | | 29 | | 41 | | 41 | |
| 11 years | 11 | | 11 | | 30 | | 31 | | 43 | | 43 | |
| 12 years | 13 | | 13 | | 33 | | 34 | | 48 | | 48 | |

| **GAMLSS (Box-Cox Power Exponential)** | | | | | | |
| --- | --- | --- | --- | --- | --- | --- |
| **Plank Time (seconds)** | **Beginning** | **Progressing** | **Progressing** | **Achieving** | **Achieving** | **Excelling** |
| **Girls** | **Less than** | **Lower** | **Upper** | **Lower** | **Upper** | **Above** |
| 8 years | 24.4 | 24.4 | 59.4 | 59.5 | 89.3 | 89.3 |
| 9 years | 25.2 | 25.2 | 61.4 | 61.5 | 92.2 | 92.2 |
| 10 years | 26.0 | 26.0 | 63.4 | 63.5 | 95.2 | 95.2 |
| 11 years | 26.8 | 26.8 | 65.3 | 65.4 | 98.2 | 98.2 |
| 12 years | 27.6 | 27.6 | 67.3 | 67.4 | 101.2 | 101.2 |
| **GAMLSS (Normal)** | | | | | | |
| **Plank Time (seconds)** | **Beginning** | **Progressing** | **Progressing** | **Achieving** | **Achieving** | **Excelling** |
| **Boys** | **Less than** | **Lower** | **Upper** | **Lower** | **Upper** | **Above** |
| 8 years | 12.4 | 12.4 | 72.0 | 72.1 | 101.0 | 101.0 |
| 9 years | 15.2 | 15.2 | 74.9 | 75.0 | 103.8 | 103.8 |
| 10 years | 18.1 | 18.1 | 77.7 | 77.8 | 106.7 | 106.7 |
| 11 years | 20.9 | 20.9 | 80.6 | 80.7 | 109.5 | 109.5 |
| 12 years | 23.8 | 23.8 | 83.4 | 83.5 | 112.4 | 112.4 |

| **WHO Criterion-Referenced Method** | | | | |
| --- | --- | --- | --- | --- |
| **BMI Z Scores** | **Beginning** | **Progressing** | **Achieving** | **Excelling** |
| **Girls and Boys** |  |  |  |  |
| 8-12 years | < -3.0 OR > 2.0 | ≥ -3.0 to < -2.0 | > 1.0 to ≤ 2.0 | ≥ -2.0 to ≤ 1.0 |

| **GAMLSS (Box-Cox Power Exponential)** | | | | | | | | | | | | |
| --- | --- | --- | --- | --- | --- | --- | --- | --- | --- | --- | --- | --- |
| **Waist Circumference (cm)** | | **Beginning** | | **Progressing** | | **Progressing** | | **Achieving** | | **Achieving** | | **Excelling** |
| **Girls** | | **Above** | | **Upper** | | **Lower** | | **Upper** | | **Lower** | | **Below** |
| 8 years | | 70.7 | | 70.7 | | 63.6 | | 63.5 | | 52.9 | | 52.9 |
| 9 years | | 73.3 | | 73.3 | | 65.9 | | 65.8 | | 54.8 | | 54.8 |
| 10 years | | 75.9 | | 75.9 | | 68.3 | | 68.2 | | 56.8 | | 56.8 |
| 11 years | | 78.5 | | 78.5 | | 70.6 | | 70.5 | | 58.7 | | 58.7 |
| 12 years | | 81.1 | | 81.1 | | 73.0 | | 72.9 | | 60.7 | | 60.7 |
| **GAMLSS (Box-Cox Power Exponential)** | | | | | | | | | | | | |
| **Waist Circumference (cm)** | **Beginning** | | **Progressing** | | **Progressing** | | **Achieving** | | **Achieving** | | **Excelling** | |
| **Boys** | **Above** | | **Upper** | | **Lower** | | **Upper** | | **Lower** | | **Below** | |
| 8 years | 70.2 | | 70.2 | | 63.1 | | 63.0 | | 53.4 | | 53.4 | |
| 9 years | 72.8 | | 72.8 | | 65.4 | | 65.3 | | 55.4 | | 55.4 | |
| 10 years | 75.3 | | 75.3 | | 67.7 | | 67.6 | | 57.3 | | 57.3 | |
| 11 years | 77.9 | | 77.9 | | 70.1 | | 70.0 | | 59.3 | | 59.3 | |
| 12 years | 80.5 | | 80.5 | | 72.4 | | 72.3 | | 61.3 | | 61.3 | |

| **GAMLSS (Box-Cox Power Exponential)** | | | | | | | | | | | | |
| --- | --- | --- | --- | --- | --- | --- | --- | --- | --- | --- | --- | --- |
| **CAMSA Max Score (out of 28)** | | **Beginning** | | **Progressing** | | **Progressing** | | **Achieving** | | **Achieving** | | **Excelling** |
| **Girls** | | **Less than** | | **Lower** | | **Upper** | | **Lower** | | **Upper** | | **Above** |
| 8 years | | 15 | | 15 | | 20 | | 21 | | 21 | | 21 |
| 9 years | | 16 | | 16 | | 21 | | 22 | | 22 | | 22 |
| 10 years | | 17 | | 17 | | 22 | | 23 | | 23 | | 23 |
| 11 years | | 17 | | 17 | | 22 | | 23 | | 24 | | 24 |
| 12 years | | 18 | | 18 | | 23 | | 24 | | 25 | | 25 |
| **GAMLSS (Box-Cox-Cole-Green)** | | | | | | | | | | | | |
| **CAMSA Max Score (out of 28)** | **Beginning** | | **Progressing** | | **Progressing** | | **Achieving** | | **Achieving** | | **Excelling** | |
| **Boys** | **Less than** | | **Lower** | | **Upper** | | **Lower** | | **Upper** | | **Above** | |
| 8 years | 16 | | 16 | | 21 | | 22 | | 23 | | 23 | |
| 9 years | 17 | | 17 | | 22 | | 23 | | 23 | | 23 | |
| 10 years | 17 | | 17 | | 22 | | 23 | | 24 | | 24 | |
| 11 years | 18 | | 18 | | 23 | | 24 | | 25 | | 25 | |
| 12 years | 18 | | 18 | | 24 | | 25 | | 26 | | 26 | |

Note: The disparity in scoring between the two methods is owing, in part, to the fact that with the GAMLSS method, the CAMSA max score was analyzed in raw form (not multiplied by 1.5).

BMI: body mass index; CAMSA: Canadian Agility and Movement Skill Assessment; GAMLSS: generalized additive models for location, scale, and shape; PACER: Progressive Aerobic Cardiovascular Endurance Run; WHO: World Health Organization

| **Semi-Criterion/GAMLSS (Box-Cox Power Exponential)** | | | | | | |
| --- | --- | --- | --- | --- | --- | --- |
| **Average Daily Step Counts** | **Beginning** | **Progressing** | **Progressing** | **Achieving** | **Achieving** | **Excelling** |
| **Girls** | **Less than** | **Lower** | **Upper** | **Lower** | **Upper** | **Above** |
| 8 years | 8059 | 8059 | 11999 | 12000 | 15643 | 15643 |
| 9 years | 7814 | 7814 | 11999 | 12000 | 15168 | 15168 |
| 10 years | 7569 | 7569 | 11999 | 12000 | 14692 | 14692 |
| 11 years | 7324 | 7324 | 11999 | 12000 | 14217 | 14217 |
| 12 years | 7079 | 7079 | 11999 | 12000 | 13742 | 13742 |
| **Semi-Criterion/GAMLSS (Box-Cox-Cole-Green)** | | | | | | |
| **Average Daily Step Counts** | **Beginning** | **Progressing** | **Progressing** | **Achieving** | **Achieving** | **Excelling** |
| **Boys** | **Less than** | **Lower** | **Upper** | **Lower** | **Upper** | **Above** |
| 8 years | 8892 | 8892 | 11999 | 12000 | 17980 | 17980 |
| 9 years | 8655 | 8655 | 11999 | 12000 | 17500 | 17500 |
| 10 years | 8417 | 8417 | 11999 | 12000 | 17020 | 17020 |
| 11 years | 8180 | 8180 | 11999 | 12000 | 16539 | 16539 |
| 12 years | 7942 | 7942 | 11999 | 12000 | 16059 | 16059 |

| **GAMLSS (Normal)** | | | | | | |
| --- | --- | --- | --- | --- | --- | --- |
| **CSAPPA Adequacy (out of 28)** | **Beginning** | **Progressing** | **Progressing** | **Achieving** | **Achieving** | **Excelling** |
| **Girls** | **Less than** | **Lower** | **Upper** | **Lower** | **Upper** | **Above** |
| 8 years | 17 | 17 | 23 | 24 | 26 | 26 |
| 9 years | 17 | 17 | 23 | 24 | 26 | 26 |
| 10 years | 17 | 17 | 23 | 24 | 26 | 26 |
| 11 years | 17 | 17 | 23 | 24 | 26 | 26 |
| 12 years | 17 | 17 | 23 | 24 | 26 | 26 |
| **GAMLSS (Box-Cox-Cole-Green)** | | | | | | |
| **CSAPPA Adequacy (out of 28)** | **Beginning** | **Progressing** | **Progressing** | **Achieving** | **Achieving** | **Excelling** |
| **Boys** | **Less than** | **Lower** | **Upper** | **Lower** | **Upper** | **Above** |
| 8 years | 18 | 18 | 24 | 25 | 26 | 26 |
| 9 years | 19 | 19 | 24 | 25 | 26 | 26 |
| 10 years | 19 | 19 | 24 | 25 | 26 | 26 |
| 11 years | 19 | 19 | 25 | 26 | 27 | 27 |
| 12 years | 19 | 19 | 25 | 26 | 27 | 27 |

| **GAMLSS (Normal)** | | | | | | |
| --- | --- | --- | --- | --- | --- | --- |
| **CSAPPA Predilection (out of 36)** | **Beginning** | **Progressing** | **Progressing** | **Achieving** | **Achieving** | **Excelling** |
| **Girls** | **Less than** | **Lower** | **Upper** | **Lower** | **Upper** | **Above** |
| 8 years | 23 | 23 | 31 | 32 | 35 | 35 |
| 9 years | 23 | 23 | 31 | 32 | 35 | 35 |
| 10 years | 23 | 23 | 31 | 32 | 35 | 35 |
| 11 years | 23 | 23 | 31 | 32 | 35 | 35 |
| 12 years | 23 | 23 | 31 | 32 | 34 | 34 |
| **GAMLSS (Box-Cox-Cole-Green)** | | | | | | |
| **CSAPPA Predilection (out of 36)** | **Beginning** | **Progressing** | **Progressing** | **Achieving** | **Achieving** | **Excelling** |
| **Boys** | **Less than** | **Lower** | **Upper** | **Lower** | **Upper** | **Above** |
| 8 years | 24 | 24 | 32 | 33 | 34 | 34 |
| 9 years | 24 | 24 | 32 | 33 | 34 | 34 |
| 10 years | 24 | 24 | 32 | 33 | 35 | 35 |
| 11 years | 24 | 24 | 32 | 33 | 35 | 35 |
| 12 years | 24 | 24 | 32 | 33 | 35 | 35 |

| **GAMLSS (Box-Cox Power Exponential)** | | | | | | |
| --- | --- | --- | --- | --- | --- | --- |
| **Benefits to Barriers Ratio (out of 35)** | **Beginning** | **Progressing** | **Progressing** | **Achieving** | **Achieving** | **Excelling** |
| **Girls** | **Less than** | **Lower** | **Upper** | **Lower** | **Upper** | **Above** |
| 8 years | 7 | 7 | 19 | 20 | 25 | 25 |
| 9 years | 7 | 7 | 18 | 19 | 24 | 24 |
| 10 years | 7 | 7 | 18 | 19 | 24 | 24 |
| 11 years | 7 | 7 | 18 | 19 | 24 | 24 |
| 12 years | 7 | 7 | 18 | 19 | 24 | 24 |
| **GAMLSS (Box-Cox Power Exponential)** | | | | | | |
| **Benefits to Barriers Ratio (out of 35)** | **Beginning** | **Progressing** | **Progressing** | **Achieving** | **Achieving** | **Excelling** |
| **Boys** | **Less than** | **Lower** | **Upper** | **Lower** | **Upper** | **Above** |
| 8 years | 8 | 8 | 21 | 22 | 27 | 27 |
| 9 years | 7 | 7 | 21 | 22 | 27 | 27 |
| 10 years | 7 | 7 | 21 | 22 | 27 | 27 |
| 11 years | 7 | 7 | 20 | 21 | 27 | 27 |
| 12 years | 7 | 7 | 20 | 21 | 26 | 26 |

| **GAMLSS (Box-Cox-Cole-Green)** | | | | | | |
| --- | --- | --- | --- | --- | --- | --- |
| **How Active Compared to Others**  **(out of 10)** | **Beginning** | **Progressing** | **Progressing** | **Achieving** | **Achieving** | **Excelling** |
| **Girls** | **Less than** | **Lower** | **Upper** | **Lower** | **Upper** | **Above** |
| 8 years | 5 | 5 | 8 | 9 | 9 | 9 |
| 9 years | 5 | 5 | 8 | 9 | 9 | 9 |
| 10 years | 5 | 5 | 8 | 9 | 9 | 9 |
| 11 years | 5 | 5 | 8 | 9 | 9 | 9 |
| 12 years | 5 | 5 | 8 | 9 | 9 | 9 |
| **GAMLSS (Box-Cox-Cole-Green)** | | | | | | |
| **How Active Compared to Others**  **(out of 10)** | **Beginning** | **Progressing** | **Progressing** | **Achieving** | **Achieving** | **Excelling** |
| **Boys** | **Less than** | **Lower** | **Upper** | **Lower** | **Upper** | **Above** |
| 8 years | 6 | 6 | 8 | 9 | 9 | 9 |
| 9 years | 5 | 5 | 8 | 9 | 9 | 9 |
| 10 years | 5 | 5 | 8 | 9 | 9 | 9 |
| 11 years | 5 | 5 | 8 | 9 | 9 | 9 |
| 12 years | 5 | 5 | 8 | 9 | 9 | 9 |

Note: The cut-points for “Achieving Upper” and “Excelling Above” in 8- to 10-year-olds are 10, which is the maximum score for this variable. Therefore, these cut-points were downgraded to 9. This also required the cut-points for “Progressing Upper” in 8- to 9-year-olds to be downgraded to 8.

| **GAMLSS (Normal)** | | | | | | |
| --- | --- | --- | --- | --- | --- | --- |
| **Skill Level Compared to Others**  **(out of 10)** | **Beginning** | **Progressing** | **Progressing** | **Achieving** | **Achieving** | **Excelling** |
| **Girls** | **Less than** | **Lower** | **Upper** | **Lower** | **Upper** | **Above** |
| 8 years | 5 | 5 | 8 | 9 | 9 | 9 |
| 9 years | 5 | 5 | 8 | 9 | 9 | 9 |
| 10 years | 4 | 4 | 7 | 8 | 9 | 9 |
| 11 years | 4 | 4 | 7 | 8 | 9 | 9 |
| 12 years | 4 | 4 | 7 | 8 | 9 | 9 |
| **GAMLSS (Box-Cox-Cole-Green)** | | | | | | |
| **Skill Level Compared to Others**  **(out of 10)** | **Beginning** | **Progressing** | **Progressing** | **Achieving** | **Achieving** | **Excelling** |
| **Boys** | **Less than** | **Lower** | **Upper** | **Lower** | **Upper** | **Above** |
| 8 years | 5 | 5 | 8 | 9 | 9 | 9 |
| 9 years | 5 | 5 | 8 | 9 | 9 | 9 |
| 10 years | 5 | 5 | 8 | 9 | 9 | 9 |
| 11 years | 5 | 5 | 8 | 9 | 9 | 9 |
| 12 years | 5 | 5 | 8 | 9 | 9 | 9 |

Note: The cut-points for “Achieving Upper” and “Excelling Above” in 8- to 9-year-olds are 10, which is the maximum score for this variable. Therefore, these cut-points were downgraded to 9.

| **Semi-Criterion/GAMLSS (Box-Cox Power Exponential)** | | | | | | |
| --- | --- | --- | --- | --- | --- | --- |
| **Average Daily Screen Time (hours)** | **Beginning** | **Progressing** | **Progressing** | **Achieving** | **Achieving** | **Excelling** |
| **Girls** | **Above** | **Lower** | **Upper** | **Lower** | **Upper** | **Below** |
| 8 years | 3.1 | 2.1 | 3.1 | 0.6 | 2.0 | 0.6 |
| 9 years | 3.2 | 2.1 | 3.2 | 0.6 | 2.0 | 0.6 |
| 10 years | 3.5 | 2.1 | 3.5 | 0.7 | 2.0 | 0.7 |
| 11 years | 3.9 | 2.1 | 3.9 | 0.8 | 2.0 | 0.8 |
| 12 years | 4.3 | 2.1 | 4.3 | 0.9 | 2.0 | 0.9 |
| **Semi-Criterion/GAMLSS (Box-Cox Power Exponential)** | | | | | | |
| **Average Daily Screen Time (hours)** | **Beginning** | **Progressing** | **Progressing** | **Achieving** | **Achieving** | **Excelling** |
| **Boys** | **Above** | **Lower** | **Upper** | **Lower** | **Upper** | **Below** |
| 8 years | 4.3 | 2.1 | 4.3 | 0.9 | 2.0 | 0.9 |
| 9 years | 4.5 | 2.1 | 4.5 | 0.9 | 2.0 | 0.9 |
| 10 years | 4.6 | 2.1 | 4.6 | 1.0 | 2.0 | 1.0 |
| 11 years | 4.8 | 2.1 | 4.8 | 1.0 | 2.0 | 1.0 |
| 12 years | 5.0 | 2.1 | 5.0 | 1.0 | 2.0 | 1.0 |

| **Semi-Criterion/GAMLSS (Box-Cox-Cole-Green)** | | | | | | |
| --- | --- | --- | --- | --- | --- | --- |
| **Self-Reported Physical Activity**  **(days per week ≥ 60 minutes)** | **Beginning** | **Progressing** | **Progressing** | **Achieving** | **Achieving** | **Excelling** |
| **Girls** | **Below** | **Lower** | **Upper** | **Lower** | **Upper** | **Above** |
| 8 years | 4 | 4 | 4 | 5 | 6 | 6 |
| 9 years | 3 | 3 | 4 | 5 | 6 | 6 |
| 10 years | 3 | 3 | 4 | 5 | 6 | 6 |
| 11 years | 3 | 3 | 4 | 5 | 6 | 6 |
| 12 years | 3 | 3 | 4 | 5 | 6 | 6 |
| **Semi-Criterion/GAMLSS (Box-Cox-Cole-Green)** | | | | | | |
| **Self-Reported Physical Activity**  **(days per week ≥ 60 minutes)** | **Beginning** | **Progressing** | **Progressing** | **Achieving** | **Achieving** | **Excelling** |
| **Boys** | **Below** | **Lower** | **Upper** | **Lower** | **Upper** | **Above** |
| 8 years | 4 | 4 | 4 | 5 | 6 | 6 |
| 9 years | 4 | 4 | 4 | 5 | 6 | 6 |
| 10 years | 4 | 4 | 4 | 5 | 6 | 6 |
| 11 years | 4 | 4 | 4 | 5 | 6 | 6 |
| 12 years | 4 | 4 | 4 | 5 | 6 | 6 |

| **GAMLSS (Box-Cox Power Exponential)** | | | | | | |
| --- | --- | --- | --- | --- | --- | --- |
| **Average Daily Non-Screen Sedentary Time (hours)** | **Beginning** | **Progressing** | **Progressing** | **Achieving** | **Achieving** | **Excelling** |
| **Girls** | **Above** | **Lower** | **Upper** | **Lower** | **Upper** | **Below** |
| 8 years | 2.9 | 2.9 | 2.0 | 1.9 | 0.6 | 0.6 |
| 9 years | 3.0 | 3.0 | 2.0 | 1.9 | 0.6 | 0.6 |
| 10 years | 3.1 | 3.1 | 2.1 | 2.0 | 0.6 | 0.6 |
| 11 years | 3.2 | 3.2 | 2.2 | 2.1 | 0.6 | 0.6 |
| 12 years | 3.5 | 3.5 | 2.4 | 2.3 | 0.7 | 0.7 |
| **GAMLSS (Box-Cox Power Exponential)** | | | | | | |
| **Average Daily Non-Screen Sedentary Time (hours)** | **Beginning** | **Progressing** | **Progressing** | **Achieving** | **Achieving** | **Excelling** |
| **Boys** | **Above** | **Lower** | **Upper** | **Lower** | **Upper** | **Below** |
| 8 years | 2.9 | 2.9 | 1.9 | 1.8 | 0.5 | 0.5 |
| 9 years | 3.0 | 3.0 | 2.0 | 1.9 | 0.5 | 0.5 |
| 10 years | 3.1 | 3.1 | 2.0 | 1.9 | 0.6 | 0.6 |
| 11 years | 3.2 | 3.2 | 2.1 | 2.0 | 0.6 | 0.6 |
| 12 years | 3.3 | 3.3 | 2.2 | 2.1 | 0.6 | 0.6 |

| **GAMLSS (Box-Cox Power Exponential)** | | | | | | |
| --- | --- | --- | --- | --- | --- | --- |
| **Musculoskeletal Fitness Score (out of 42)** | **Beginning** | **Progressing** | **Progressing** | **Achieving** | **Achieving** | **Excelling** |
| **Girls** | **Below** | **Lower** | **Upper** | **Lower** | **Upper** | **Above** |
| 8 years | 12 | 12 | 20 | 21 | 24 | 24 |
| 9 years | 13 | 13 | 22 | 23 | 26 | 26 |
| 10 years | 14 | 14 | 23 | 24 | 28 | 28 |
| 11 years | 15 | 15 | 25 | 26 | 30 | 30 |
| 12 years | 17 | 17 | 27 | 28 | 33 | 33 |
| **GAMLSS (Box-Cox Power Exponential)** | | | | | | |
| **Musculoskeletal Fitness Score (out of 42)** | **Beginning** | **Progressing** | **Progressing** | **Achieving** | **Achieving** | **Excelling** |
| **Boys** | **Below** | **Lower** | **Upper** | **Lower** | **Upper** | **Above** |
| 8 years | 11 | 11 | 19 | 20 | 24 | 24 |
| 9 years | 13 | 13 | 22 | 23 | 26 | 26 |
| 10 years | 14 | 14 | 23 | 24 | 29 | 29 |
| 11 years | 14 | 14 | 24 | 25 | 29 | 29 |
| 12 years | 16 | 16 | 27 | 28 | 33 | 33 |

| **GAMLSS (Box-Cox-Cole-Green)** | | | | | | |
| --- | --- | --- | --- | --- | --- | --- |
| **Body Composition Score**  **(out of 34)** | **Beginning** | **Progressing** | **Progressing** | **Achieving** | **Achieving** | **Excelling** |
| **Girls** | **Below** | **Lower** | **Upper** | **Lower** | **Upper** | **Above** |
| 8 years | 21 | 21 | 32 | 33 | 33 | 33 |
| 9 years | 21 | 21 | 31 | 32 | 33 | 33 |
| 10 years | 21 | 21 | 30 | 31 | 33 | 33 |
| 11 years | 20 | 20 | 29 | 30 | 33 | 33 |
| 12 years | 19 | 19 | 28 | 29 | 32 | 32 |
| **GAMLSS (Box-Cox-Cole-Green)** | | | | | | |
| **Body Composition Score**  **(out of 34)** | **Beginning** | **Progressing** | **Progressing** | **Achieving** | **Achieving** | **Excelling** |
| **Boys** | **Below** | **Lower** | **Upper** | **Lower** | **Upper** | **Above** |
| 8 years | 21 | 21 | 32 | 33 | 33 | 33 |
| 9 years | 20 | 20 | 31 | 32 | 33 | 33 |
| 10 years | 20 | 20 | 30 | 31 | 33 | 33 |
| 11 years | 19 | 19 | 29 | 30 | 33 | 33 |
| 12 years | 19 | 19 | 28 | 29 | 32 | 32 |

Note: The maximum score is 34 so the predicted cut-points for “Achieving Upper” and “Excelling Above” in 8- to 10-year-olds (34-36) are problematic. Therefore, cut-points downgraded to 33.

| **GAMLSS (Box-Cox Power Exponential)** | | | | | | |
| --- | --- | --- | --- | --- | --- | --- |
| **Physical Competence Score (out of 32)** | **Beginning** | **Progressing** | **Progressing** | **Achieving** | **Achieving** | **Excelling** |
| **Girls** | **Below** | **Lower** | **Upper** | **Lower** | **Upper** | **Above** |
| 8 years | 14.1 | 14.1 | 19.2 | 19.3 | 21.7 | 21.7 |
| 9 years | 14.6 | 14.6 | 19.8 | 19.9 | 22.3 | 22.3 |
| 10 years | 15.0 | 15.0 | 20.4 | 20.5 | 23.0 | 23.0 |
| 11 years | 15.5 | 15.5 | 21.1 | 21.2 | 23.8 | 23.8 |
| 12 years | 16.2 | 16.2 | 22.1 | 22.2 | 24.9 | 24.9 |
| **GAMLSS (Box-Cox Power Exponential)** | | | | | | |
| **Physical Competence Score (out of 32)** | **Beginning** | **Progressing** | **Progressing** | **Achieving** | **Achieving** | **Excelling** |
| **Boys** | **Below** | **Lower** | **Upper** | **Lower** | **Upper** | **Above** |
| 8 years | 14.3 | 14.3 | 20.7 | 20.8 | 23.5 | 23.5 |
| 9 years | 14.6 | 14.6 | 21.2 | 21.3 | 24.0 | 24.0 |
| 10 years | 14.9 | 14.9 | 21.6 | 21.7 | 24.5 | 24.5 |
| 11 years | 15.3 | 15.3 | 22.2 | 22.3 | 25.2 | 25.2 |
| 12 years | 15.9 | 15.9 | 23.0 | 23.1 | 26.1 | 26.1 |

| **GAMLSS (Box-Cox Power Exponential)** | | | | | | |
| --- | --- | --- | --- | --- | --- | --- |
| **Daily Behaviour Score (out of 32)** | **Beginning** | **Progressing** | **Progressing** | **Achieving** | **Achieving** | **Excelling** |
| **Girls** | **Below** | **Lower** | **Upper** | **Lower** | **Upper** | **Above** |
| 8 years | 11.5 | 11.5 | 23.0 | 23.1 | 27.9 | 27.9 |
| 9 years | 11.4 | 11.4 | 22.9 | 23.0 | 27.8 | 27.8 |
| 10 years | 11.2 | 11.2 | 22.5 | 22.6 | 27.4 | 27.4 |
| 11 years | 10.8 | 10.8 | 21.8 | 21.9 | 26.4 | 26.4 |
| 12 years | 10.8 | 10.8 | 21.7 | 21.8 | 26.3 | 26.3 |
| **GAMLSS (Box-Cox Power Exponential)** | | | | | | |
| **Daily Behaviour Score (out of 32)** | **Beginning** | **Progressing** | **Progressing** | **Achieving** | **Achieving** | **Excelling** |
| **Boys** | **Below** | **Lower** | **Upper** | **Lower** | **Upper** | **Above** |
| 8 years | 9.4 | 9.4 | 23.8 | 23.9 | 28.7 | 28.7 |
| 9 years | 9.4 | 9.4 | 23.8 | 23.9 | 28.7 | 28.7 |
| 10 years | 9.4 | 9.4 | 23.8 | 23.9 | 28.7 | 28.7 |
| 11 years | 9.4 | 9.4 | 23.8 | 23.9 | 28.7 | 28.7 |
| 12 years | 9.4 | 9.4 | 23.8 | 23.9 | 28.7 | 28.7 |

| **GAMLSS (Box-Cox Power Exponential)** | | | | | | |
| --- | --- | --- | --- | --- | --- | --- |
| **Motivation and Confidence Score (out of 18)** | **Beginning** | **Progressing** | **Progressing** | **Achieving** | **Achieving** | **Excelling** |
| **Girls** | **Below** | **Lower** | **Upper** | **Lower** | **Upper** | **Above** |
| 8 years | 9.7 | 9.7 | 13.4 | 13.5 | 14.9 | 14.9 |
| 9 years | 9.7 | 9.7 | 13.5 | 13.6 | 14.9 | 14.9 |
| 10 years | 9.7 | 9.7 | 13.5 | 13.6 | 14.9 | 14.9 |
| 11 years | 9.7 | 9.7 | 13.5 | 13.6 | 15.0 | 15.0 |
| 12 years | 9.8 | 9.8 | 13.5 | 13.6 | 15.0 | 15.0 |
| **GAMLSS (Box-Cox Power Exponential)** | | | | | | |
| **Motivation and Confidence Score (out of 18)** | **Beginning** | **Progressing** | **Progressing** | **Achieving** | **Achieving** | **Excelling** |
| **Boys** | **Below** | **Lower** | **Upper** | **Lower** | **Upper** | **Above** |
| 8 years | 9.8 | 9.8 | 13.8 | 13.9 | 15.2 | 15.2 |
| 9 years | 10.0 | 10.0 | 14.0 | 14.1 | 15.4 | 15.4 |
| 10 years | 10.1 | 10.1 | 14.1 | 14.2 | 15.6 | 15.6 |
| 11 years | 10.1 | 10.1 | 14.2 | 14.3 | 15.6 | 15.6 |
| 12 years | 10.1 | 10.1 | 14.2 | 14.3 | 15.7 | 15.7 |

| **GAMLSS (Box-Cox Power Exponential)** | | | | | | |
| --- | --- | --- | --- | --- | --- | --- |
| **Knowledge and Understanding Score (out of 18)** | **Beginning** | **Progressing** | **Progressing** | **Achieving** | **Achieving** | **Excelling** |
| **Girls** | **Below** | **Lower** | **Upper** | **Lower** | **Upper** | **Above** |
| 8 years | 8.6 | 8.6 | 11.9 | 12.0 | 13.2 | 13.2 |
| 9 years | 9.0 | 9.0 | 12.5 | 12.6 | 13.9 | 13.9 |
| 10 years | 9.5 | 9.5 | 13.2 | 13.3 | 14.6 | 14.6 |
| 11 years | 9.9 | 9.9 | 13.7 | 13.8 | 15.1 | 15.1 |
| 12 years | 10.1 | 10.1 | 14.0 | 14.1 | 15.4 | 15.4 |
| **GAMLSS (Box-Cox Power Exponential)** | | | | | | |
| **Knowledge and Understanding Score (out of 18)** | **Beginning** | **Progressing** | **Progressing** | **Achieving** | **Achieving** | **Excelling** |
| **Boys** | **Below** | **Lower** | **Upper** | **Lower** | **Upper** | **Above** |
| 8 years | 8.0 | 8.0 | 11.6 | 11.7 | 12.9 | 12.9 |
| 9 years | 8.5 | 8.5 | 12.3 | 12.4 | 13.7 | 13.7 |
| 10 years | 9.0 | 9.0 | 13.0 | 13.1 | 14.5 | 14.5 |
| 11 years | 9.4 | 9.4 | 13.5 | 13.6 | 15.1 | 15.1 |
| 12 years | 9.5 | 9.5 | 13.7 | 13.8 | 15.3 | 15.3 |

| **GAMLSS (Box-Cox Power Exponential)** | | | | | | |
| --- | --- | --- | --- | --- | --- | --- |
| **Overall Physical Literacy Score (out of 100)** | **Beginning** | **Progressing** | **Progressing** | **Achieving** | **Achieving** | **Excelling** |
| **Girls** | **Below** | **Lower** | **Upper** | **Lower** | **Upper** | **Above** |
| 8 years | 49.6 | 49.6 | 64.8 | 64.9 | 71.7 | 71.7 |
| 9 years | 50.6 | 50.6 | 66.1 | 66.2 | 73.1 | 73.1 |
| 10 years | 51.2 | 51.2 | 66.8 | 66.9 | 73.9 | 73.9 |
| 11 years | 51.3 | 51.3 | 67.0 | 67.1 | 74.1 | 74.1 |
| 12 years | 52.1 | 52.1 | 68.1 | 68.2 | 75.3 | 75.3 |
| **GAMLSS (Box-Cox Power Exponential)** | | | | | | |
| **Overall Physical Literacy Score (out of 100)** | **Beginning** | **Progressing** | **Progressing** | **Achieving** | **Achieving** | **Excelling** |
| **Boys** | **Below** | **Lower** | **Upper** | **Lower** | **Upper** | **Above** |
| 8 years | 47.3 | 47.3 | 65.3 | 65.4 | 72.7 | 72.7 |
| 9 years | 48.8 | 48.8 | 67.4 | 67.5 | 75.0 | 75.0 |
| 10 years | 49.8 | 49.8 | 68.7 | 68.8 | 76.4 | 76.4 |
| 11 years | 50.2 | 50.2 | 69.3 | 69.4 | 77.1 | 77.1 |
| 12 years | 51.6 | 51.6 | 71.1 | 71.2 | 79.1 | 79.1 |
