# Supplementary material for: Physical literacy levels of Canadian children aged 8–12 years: descriptive and normative results from the RBC Learn to Play–CAPL project
Source: BMC Public Health. 2018 Oct 2;18(Suppl 2):1036. doi: 10.1186/s12889-018-5891-x (PMC6167776; doi:10.1186/s12889-018-5891-x)
Supplement: Supplementary file 2 — RBC Learn to Play–CAPL descriptive statistics stratified by gender and age. (DOCX 50 kb) [file 12889_2018_5891_MOESM2_ESM.docx]

**Table 1.** RBC Learn to Play–CAPL descriptive statistics for boys by age.

| Variable | 8 years | | 9 years | | 10 years | | 11 years | | 12 years | |
| --- | --- | --- | --- | --- | --- | --- | --- | --- | --- | --- |
|  | n | Mean ± SD | n | Mean ± SD | n | Mean ± SD | n | Mean ± SD | n | Mean ± SD |
| Age (years) | 555 | 8.5 ± 0.3 | 992 | 9.5 ± 0.3 | 1248 | 10.5 ± 0.3 | 1584 | 11.5 ± 0.3 | 566 | 12.3 ± 0.2 |
| Physical Competence score (/32) | 534 | 18.9 ± 4.2 | 938 | 19.3 ± 4.4 | 1181 | 19.7 ± 4.6 | 1485 | 20.4 ± 4.8 | 524 | 21.2 ± 4.8 |
| Sit-and-reach max score (cm) | 539 | 27.0 ± 6.8 | 952 | 26.4 ± 7.6 | 1202 | 25.5 ± 7.5 | 1514 | 24.7 ± 7.6 | 534 | 24.1 ± 7.8 |
| Total handgrip strength (kg) | 540 | 27.1 ± 6.2 | 952 | 30.7 ± 7.2 | 1216 | 33.3 ± 8.1 | 1515 | 37.5 ± 9.2 | 537 | 42.8 ± 11.0 |
| PACER (20m laps) | 522 | 22.8 ± 14.3 | 933 | 24.0 ± 14.3 | 1181 | 25.0 ± 14.7 | 1496 | 27.2 ± 16.8 | 525 | 30.3 ± 18.1 |
| Plank time (sec) | 537 | 53.8 ± 38.1 | 944 | 61.0 ± 46.4 | 1202 | 61.1 ± 44.8 | 1513 | 65.6 ± 47.2 | 531 | 65.5 ± 38.3 |
| Body mass index (kg/m^2^) | 534 | 17.2 ± 2.7 | 943 | 18.2 ± 3.5 | 1202 | 18.9 ± 3.8 | 1490 | 19.7 ± 4.1 | 520 | 19.9 ± 4.3 |
| Waist circumference (cm) | 533 | 61.0 ± 7.2 | 928 | 64.3 ± 9.4 | 1185 | 67.2 ± 10.6 | 1482 | 70.5 ± 11.7 | 522 | 71.3 ± 11.7 |
| CAMSA max score (/28) | 539 | 18.6 ± 4.2 | 940 | 20.0 ± 3.8 | 1186 | 20.9 ± 3.8 | 1510 | 22.0 ± 3.6 | 523 | 22.5 ± 3.6 |
| Daily Behaviour score (/32) | 542 | 19.1 ± 7.7 | 971 | 19.8 ± 7.7 | 1228 | 18.6 ± 7.7 | 1573 | 18.0 ± 8.0 | 552 | 18.1 ± 8.6 |
| Daily steps taken | 348 | 12890 ± 4264 | 583 | 13160 ± 4292 | 813 | 12344 ± 4229 | 983 | 11834 ± 4091 | 335 | 11833 ± 4475 |
| Physical activity guideline adherence (days/week) | 543 | 4.9 ± 2.2 | 973 | 5.0 ± 2.1 | 1229 | 5.1 ± 1.9 | 1571 | 5.0 ± 1.9 | 552 | 5.0 ± 1.9 |
| Daily screen time (hrs) | 539 | 2.6 ± 2.2 | 963 | 2.6 ± 2.2 | 1227 | 2.7 ± 2.0 | 1572 | 2.9 ± 2.0 | 551 | 2.9 ± 2.0 |
| Daily non-screen time (hrs) | 537 | 1.4 ± 1.3 | 970 | 1.5 ± 1.3 | 1228 | 1.6 ± 1.3 | 1573 | 1.7 ± 1.3 | 554 | 1.7 ± 1.3 |
| Motivation and Confidence score (/18) | 524 | 12.4 ± 2.6 | 946 | 12.7 ± 2.6 | 1204 | 12.9 ± 2.7 | 1546 | 12.7 ± 3.0 | 557 | 12.7 ± 3.1 |
| Adequacy (/28) | 526 | 21.9 ± 4.1 | 948 | 22.2 ± 4.0 | 1201 | 22.6 ± 4.2 | 1546 | 22.3 ± 4.4 | 557 | 22.7 ± 4.5 |
| Predilection (/36) | 526 | 28.3 ± 5.8 | 948 | 29.0 ± 5.8 | 1201 | 29.3 ± 5.9 | 1546 | 28.9 ± 6.2 | 557 | 29.0 ± 6.5 |
| Benefits-to-barriers ratio (/4) | 536 | 1.5 ± 1.3 | 962 | 1.6 ± 1.2 | 1227 | 1.7 ± 1.2 | 1569 | 1.6 ± 1.2 | 560 | 1.6 ± 1.2 |
| Physical activity level compared to peers (/10) | 545 | 7.4 ± 2.3 | 976 | 7.5 ± 2.2 | 1239 | 7.3 ± 2.1 | 1580 | 7.1 ± 2.1 | 565 | 7.1 ± 2.2 |
| Skill level compared to peers (/10) | 547 | 7.2 ± 2.6 | 975 | 7.2 ± 2.4 | 1239 | 7.1 ± 2.4 | 1580 | 6.8 ± 2.3 | 564 | 7.0 ± 2.3 |
| Knowledge and Understanding score (/18) | 540 | 10.2 ± 2.7 | 970 | 11.0 ± 2.7 | 1228 | 12.0 ± 2.7 | 1577 | 12.5 ± 2.7 | 554 | 12.7 ± 2.6 |
| Minutes of daily MVPA (/1) | 545 | 0.5 ± 0.5 | 975 | 0.6 ± 0.5 | 1236 | 0.7 ± 0.5 | 1580 | 0.7 ± 0.5 | 555 | 0.7 ± 0.4 |
| Minutes of daily screen time (/1) | 546 | 0.1 ± 0.3 | 976 | 0.1 ± 0.3 | 1234 | 0.2 ± 0.4 | 1580 | 0.2 ± 0.4 | 556 | 0.2 ± 0.4 |
| Cardiorespiratory fitness definition (/1) | 545 | 0.4 ± 0.5 | 970 | 0.4 ± 0.5 | 1232 | 0.5 ± 0.5 | 1578 | 0.6 ± 0.5 | 556 | 0.6 ± 0.5 |
| Muscular endurance definition (/1) | 547 | 0.6 ± 0.5 | 975 | 0.6 ± 0.5 | 1233 | 0.7 ± 0.5 | 1578 | 0.8 ± 0.4 | 556 | 0.8 ± 0.4 |
| Healthy definition (/5) | 550 | 3.8 ± 1.0 | 980 | 3.8 ± 1.0 | 1240 | 3.9 ± 1.0 | 1580 | 3.9 ± 1.0 | 556 | 3.9 ± 0.9 |
| Fill in the missing words (/5) | 543 | 2.8 ± 1.6 | 974 | 3.2 ± 1.5 | 1233 | 3.7 ± 1.4 | 1574 | 4.0 ± 1.4 | 555 | 4.1 ± 1.3 |
| Physical activity checklist (/1) | 550 | 0.3 ± 0.3 | 980 | 0.3 ± 0.3 | 1240 | 0.3 ± 0.3 | 1580 | 0.3 ± 0.3 | 556 | 0.3 ± 0.3 |
| How to get better at a sport skill (/1) | 540 | 0.4 ± 0.5 | 971 | 0.5 ± 0.5 | 1227 | 0.5 ± 0.5 | 1578 | 0.5 ± 0.5 | 554 | 0.5 ± 0.5 |
| How to improve physical fitness (/1) | 539 | 0.7 ± 0.5 | 974 | 0.7 ± 0.4 | 1227 | 0.8 ± 0.4 | 1578 | 0.8 ± 0.4 | 553 | 0.8 ± 0.4 |
| Preferred leisure-time activity (/1) | 545 | 0.6 ± 0.5 | 976 | 0.7 ± 0.5 | 1238 | 0.7 ± 0.5 | 1578 | 0.7 ± 0.5 | 556 | 0.7 ± 0.5 |
| Physical literacy score (/100) | 542 | 60.6 ± 12.1 | 971 | 62.7 ± 12.3 | 1227 | 63.1 ± 12.8 | 1572 | 63.5 ± 13.3 | 553 | 65.1 ± 14.5 |

CAMSA: Canadian Agility and Movement Skill Assessment; CAPL: Canadian Assessment of Physical Literacy; MVPA: moderate- to vigorous-intensity physical activity; PACER: Progressive Aerobic Cardiovascular Endurance Run; RBC: Royal Bank of Canada; SD: standard deviation

**Table 2.** RBC Learn to Play–CAPL descriptive statistics for girls by age.

| Variable | 8 years | | 9 years | | 10 years | | 11 years | | 12 years | |
| --- | --- | --- | --- | --- | --- | --- | --- | --- | --- | --- |
|  | n | Mean ± SD | n | Mean ± SD | n | Mean ± SD | n | Mean ± SD | n | Mean ± SD |
| Age (years) | 562 | 8.5 ± 0.3 | 966 | 9.5 ± 0.3 | 1240 | 10.5 ± 0.3 | 1638 | 11.5 ± 0.3 | 578 | 12.3 ± 0.2 |
| Physical Competence score (/32) | 534 | 18.0 ± 3.7 | 913 | 18.6 ± 4.1 | 1162 | 18.9 ± 4.0 | 1548 | 20.0 ± 4.0 | 529 | 20.7 ± 4.1 |
| Sit-and-reach max score (cm) | 549 | 31.2 ± 7.2 | 926 | 31.1 ± 7.8 | 1177 | 30.5 ± 8.2 | 1582 | 30.7 ± 8.7 | 548 | 31.9 ± 8.8 |
| Total handgrip strength (kg) | 547 | 25.3 ± 5.6 | 923 | 28.3 ± 6.5 | 1198 | 31.4 ± 7.7 | 1593 | 35.7 ± 9.0 | 551 | 40.1 ± 10.0 |
| PACER (20m laps) | 523 | 18.5 ± 11.1 | 900 | 19.5 ± 10.8 | 1160 | 19.8 ± 11.1 | 1536 | 22.3 ± 11.7 | 522 | 24.0 ± 12.9 |
| Plank time (sec) | 543 | 55.6 ± 40.8 | 925 | 61.7 ± 44.5 | 1189 | 59.9 ± 40.5 | 1580 | 63.1 ± 42.9 | 546 | 64.8 ± 46.8 |
| Body mass index (kg/m^2^) | 530 | 17.3 ± 2.9 | 917 | 18.4 ± 3.5 | 1176 | 19.0 ± 3.7 | 1561 | 19.5 ± 3.8 | 538 | 20.0 ± 4.0 |
| Waist circumference (cm) | 542 | 61.6 ± 8.6 | 909 | 64.5 ± 9.8 | 1164 | 67.1 ± 10.3 | 1554 | 69.2 ± 10.5 | 532 | 71.3 ± 10.4 |
| CAMSA max score (/28) | 537 | 17.4 ± 4.0 | 906 | 19.4 ± 3.8 | 1171 | 20.1 ± 3.6 | 1558 | 21.4 ± 3.4 | 523 | 22.0 ± 3.4 |
| Daily Behaviour score (/32) | 547 | 20.1 ± 7.1 | 940 | 19.6 ± 7.1 | 1233 | 18.7 ± 7.3 | 1633 | 17.5 ± 7.5 | 560 | 17.4 ± 7.9 |
| Daily steps taken | 358 | 11450 ± 3421 | 685 | 11186 ± 3528 | 914 | 10896 ± 3796 | 1180 | 10449 ± 3655 | 393 | 10141 ± 3297 |
| Physical activity guideline adherence (days/week) | 549 | 4.8 ± 2.1 | 940 | 5.0 ± 1.9 | 1232 | 4.9 ± 1.8 | 1634 | 4.8 ± 1.8 | 560 | 4.9 ± 1.8 |
| Daily screen time (hrs) | 547 | 1.9 ± 1.8 | 942 | 1.9 ± 1.7 | 1232 | 2.0 ± 1.7 | 1632 | 2.4 ± 1.8 | 561 | 2.5 ± 1.8 |
| Daily non-screen time (hrs) | 543 | 1.5 ± 1.3 | 944 | 1.5 ± 1.3 | 1230 | 1.7 ± 1.3 | 1633 | 1.8 ± 1.3 | 560 | 2.1 ± 1.3 |
| Motivation and Confidence score (/18) | 534 | 12.3 ± 2.4 | 927 | 12.5 ± 2.4 | 1201 | 12.2 ± 2.7 | 1615 | 12.1 ± 2.7 | 567 | 12.2 ± 2.8 |
| Adequacy (/28) | 535 | 21.5 ± 4.0 | 929 | 21.5 ± 4.2 | 1202 | 21.4 ± 4.3 | 1613 | 21.2 ± 4.3 | 566 | 21.6 ± 4.4 |
| Predilection (/36) | 535 | 28.8 ± 5.5 | 929 | 29.0 ± 5.6 | 1202 | 29.0 ± 5.9 | 1613 | 28.3 ± 5.7 | 566 | 28.5 ± 6.0 |
| Benefits-to-barriers ratio (/4) | 537 | 1.4 ± 1.2 | 938 | 1.6 ± 1.1 | 1223 | 1.5 ± 1.1 | 1624 | 1.5 ± 1.1 | 567 | 1.5 ± 1.1 |
| Physical activity level compared to peers (/10) | 557 | 7.5 ± 2.2 | 950 | 7.2 ± 2.1 | 1235 | 7.0 ± 2.0 | 1637 | 6.8 ± 1.9 | 577 | 7.0 ± 2.0 |
| Skill level compared to peers (/10) | 555 | 6.9 ± 2.3 | 950 | 6.6 ± 2.3 | 1236 | 6.3 ± 2.2 | 1636 | 6.2 ± 2.1 | 577 | 6.4 ± 2.1 |
| Knowledge and Understanding score (/18) | 552 | 10.6 ± 2.6 | 946 | 11.4 ± 2.5 | 1233 | 12.3 ± 2.6 | 1633 | 12.8 ± 2.5 | 561 | 13.0 ± 2.5 |
| Minutes of daily MVPA (/1) | 554 | 0.5 ± 0.5 | 952 | 0.5 ± 0.5 | 1235 | 0.6 ± 0.5 | 1636 | 0.7 ± 0.5 | 562 | 0.7 ± 0.5 |
| Minutes of daily screen time (/1) | 557 | 0.1 ± 0.3 | 951 | 0.1 ± 0.2 | 1234 | 0.1 ± 0.3 | 1637 | 0.2 ± 0.4 | 562 | 0.2 ± 0.4 |
| Cardiorespiratory fitness definition (/1) | 556 | 0.4 ± 0.5 | 949 | 0.5 ± 0.5 | 1234 | 0.6 ± 0.5 | 1636 | 0.7 ± 0.5 | 562 | 0.7 ± 0.5 |
| Muscular endurance definition (/1) | 555 | 0.6 ± 0.5 | 950 | 0.7 ± 0.5 | 1234 | 0.8 ± 0.4 | 1635 | 0.8 ± 0.4 | 562 | 0.8 ± 0.4 |
| Healthy definition (/5) | 557 | 4.0 ± 0.9 | 953 | 4.0 ± 0.9 | 1236 | 4.0 ± 0.9 | 1637 | 4.1 ± 0.9 | 562 | 4.0 ± 0.9 |
| Fill in the missing words (/5) | 555 | 2.9 ± 1.5 | 947 | 3.2 ± 1.4 | 1235 | 3.6 ± 1.4 | 1634 | 3.9 ± 1.4 | 561 | 4.0 ± 1.3 |
| Physical activity checklist (/1) | 557 | 0.4 ± 0.3 | 953 | 0.4 ± 0.3 | 1236 | 0.4 ± 0.3 | 1637 | 0.4 ± 0.3 | 562 | 0.4 ± 0.3 |
| How to get better at a sport skill (/1) | 552 | 0.4 ± 0.5 | 947 | 0.5 ± 0.5 | 1233 | 0.5 ± 0.5 | 1632 | 0.5 ± 0.5 | 560 | 0.6 ± 0.5 |
| How to improve physical fitness (/1) | 553 | 0.7 ± 0.5 | 947 | 0.8 ± 0.4 | 1235 | 0.8 ± 0.4 | 1633 | 0.9 ± 0.4 | 561 | 0.8 ± 0.4 |
| Preferred leisure-time activity (/1) | 553 | 0.7 ± 0.5 | 952 | 0.7 ± 0.4 | 1235 | 0.8 ± 0.4 | 1636 | 0.8 ± 0.4 | 562 | 0.8 ± 0.4 |
| Physical literacy score (/100) | 551 | 61.1 ± 10.2 | 944 | 62.1 ± 10.5 | 1229 | 62.2 ± 11.5 | 1630 | 62.4 ± 11.6 | 559 | 63.4 ± 12.4 |

CAMSA: Canadian Agility and Movement Skill Assessment; CAPL: Canadian Assessment of Physical Literacy; MVPA, moderate- to vigorous-intensity physical activity; PACER: Progressive Aerobic Cardiovascular Endurance Run; RBC: Royal Bank of Canada; SD: standard deviation
