# Supplementary material for: Physical literacy levels of Canadian children aged 8–12 years: descriptive and normative results from the RBC Learn to Play–CAPL project
Source: BMC Public Health. 2018 Oct 2;18(Suppl 2):1036. doi: 10.1186/s12889-018-5891-x (PMC6167776; doi:10.1186/s12889-018-5891-x)
Supplement: Supplementary file 3 — Percentiles for the total physical literacy score, domain scores, the individual components in the physical competence domain, and daily step counts. (DOCX 132 kb) [file 12889_2018_5891_MOESM3_ESM.docx]

**Table 1.** Percentiles of the Physical Competence score (out of 32), by gender and age, calculated with generalized additive models for location, scale, and shape (GAMLSS).

| Gender/Age | GAMLSS | Percentiles | | | | | | | | | | | | | | | | | | |
| --- | --- | --- | --- | --- | --- | --- | --- | --- | --- | --- | --- | --- | --- | --- | --- | --- | --- | --- | --- | --- |
|  |  | 5 | 10 | 15 | 20 | 25 | 30 | 35 | 40 | 45 | 50 | 55 | 60 | 65 | 70 | 75 | 80 | 85 | 90 | 95 |
| Boys |  |  |  |  |  |  |  |  |  |  |  |  |  |  |  |  |  |  |  |  |
| 8.0-8.9 | BCPE | 11.6 | 13.0 | 13.9 | 14.7 | 15.5 | 16.2 | 16.8 | 17.5 | 18.1 | 18.7 | 19.4 | 20.0 | 20.7 | 21.3 | 22.0 | 22.7 | 23.5 | 24.4 | 25.6 |
| 9.0-9.9 | BCPE | 11.9 | 13.3 | 14.3 | 15.1 | 15.8 | 16.5 | 17.2 | 17.9 | 18.5 | 19.2 | 19.8 | 20.5 | 21.2 | 21.8 | 22.5 | 23.2 | 24.0 | 25.0 | 26.2 |
| 10.0-10.9 | BCPE | 12.2 | 13.6 | 14.6 | 15.4 | 16.2 | 16.9 | 17.6 | 18.3 | 18.9 | 19.6 | 20.3 | 20.9 | 21.6 | 22.3 | 23.0 | 23.7 | 24.5 | 25.5 | 26.8 |
| 11.0-11.9 | BCPE | 12.5 | 13.9 | 15.0 | 15.8 | 16.6 | 17.4 | 18.1 | 18.8 | 19.5 | 20.1 | 20.8 | 21.5 | 22.2 | 22.9 | 23.6 | 24.4 | 25.2 | 26.2 | 27.5 |
| 12.0-12.9 | BCPE | 13.0 | 14.4 | 15.5 | 16.4 | 17.2 | 18.0 | 18.7 | 19.4 | 20.2 | 20.9 | 21.6 | 22.3 | 23.0 | 23.7 | 24.5 | 25.3 | 26.1 | 27.1 | 28.5 |
| Girls |  |  |  |  |  |  |  |  |  |  |  |  |  |  |  |  |  |  |  |  |
| 8.0-8.9 | BCPE | 11.8 | 13.0 | 13.9 | 14.6 | 15.2 | 15.7 | 16.2 | 16.7 | 17.2 | 17.7 | 18.2 | 18.7 | 19.2 | 19.8 | 20.3 | 21.0 | 21.7 | 22.6 | 23.8 |
| 9.0-9.9 | BCPE | 12.1 | 13.4 | 14.3 | 15.0 | 15.6 | 16.2 | 16.7 | 17.2 | 17.8 | 18.3 | 18.8 | 19.3 | 19.8 | 20.4 | 21.0 | 21.6 | 22.3 | 23.2 | 24.5 |
| 10.0-10.9 | BCPE | 12.5 | 13.8 | 14.7 | 15.4 | 16.1 | 16.7 | 17.2 | 17.8 | 18.3 | 18.8 | 19.3 | 19.9 | 20.4 | 21.0 | 21.6 | 22.2 | 23.0 | 23.9 | 25.3 |
| 11.0-11.9 | BCPE | 12.9 | 14.3 | 15.2 | 16.0 | 16.6 | 17.3 | 17.8 | 18.4 | 18.9 | 19.5 | 20.0 | 20.6 | 21.1 | 21.7 | 22.3 | 23.0 | 23.8 | 24.8 | 26.2 |
| 12.0-12.9 | BCPE | 13.5 | 14.9 | 15.9 | 16.7 | 17.4 | 18.0 | 18.6 | 19.2 | 19.8 | 20.3 | 20.9 | 21.5 | 22.1 | 22.7 | 23.3 | 24.1 | 24.9 | 25.9 | 27.3 |

BCPE: Box-Cox power exponential distribution; GAMLSS: generalized additive models for location, scale, and shape

**Table 2.** Percentiles of the sit-and-reach max score (cm), by gender and age, calculated with generalized additive models for location, scale, and shape (GAMLSS).

| Gender/Age | GAMLSS | Percentiles | | | | | | | | | | | | | | | | | | |
| --- | --- | --- | --- | --- | --- | --- | --- | --- | --- | --- | --- | --- | --- | --- | --- | --- | --- | --- | --- | --- |
|  |  | 5 | 10 | 15 | 20 | 25 | 30 | 35 | 40 | 45 | 50 | 55 | 60 | 65 | 70 | 75 | 80 | 85 | 90 | 95 |
| Boys |  |  |  |  |  |  |  |  |  |  |  |  |  |  |  |  |  |  |  |  |
| 8.0-8.9 | NO | 15.3 | 18.0 | 19.8 | 21.3 | 22.5 | 23.6 | 24.7 | 25.6 | 26.6 | 27.5 | 28.4 | 29.4 | 30.4 | 31.4 | 32.5 | 33.8 | 35.2 | 37.0 | 39.7 |
| 9.0-9.9 | NO | 14.5 | 17.2 | 19.0 | 20.5 | 21.7 | 22.8 | 23.9 | 24.9 | 25.8 | 26.7 | 27.7 | 28.6 | 29.6 | 30.6 | 31.7 | 33.0 | 34.4 | 36.2 | 38.9 |
| 10.0-10.9 | NO | 13.7 | 16.4 | 18.3 | 19.7 | 20.9 | 22.1 | 23.1 | 24.1 | 25.0 | 25.9 | 26.9 | 27.8 | 28.8 | 29.8 | 31.0 | 32.2 | 33.6 | 35.5 | 38.2 |
| 11.0-11.9 | NO | 13.0 | 15.7 | 17.5 | 18.9 | 20.2 | 21.3 | 22.3 | 23.3 | 24.2 | 25.2 | 26.1 | 27.0 | 28.0 | 29.1 | 30.2 | 31.4 | 32.9 | 34.7 | 37.4 |
| 12.0-12.9 | NO | 12.2 | 14.9 | 16.7 | 18.1 | 19.4 | 20.5 | 21.5 | 22.5 | 23.5 | 24.4 | 25.3 | 26.3 | 27.2 | 28.3 | 29.4 | 30.6 | 32.1 | 33.9 | 36.6 |
| Girls |  |  |  |  |  |  |  |  |  |  |  |  |  |  |  |  |  |  |  |  |
| 8.0-8.9 | BCPE | 16.6 | 19.7 | 21.7 | 23.3 | 24.7 | 26.0 | 27.2 | 28.3 | 29.4 | 30.5 | 31.5 | 32.6 | 33.7 | 34.8 | 36.0 | 37.3 | 38.8 | 40.6 | 43.1 |
| 9.0-9.9 | BCPE | 16.7 | 19.8 | 21.8 | 23.5 | 24.9 | 26.2 | 27.3 | 28.5 | 29.6 | 30.7 | 31.7 | 32.8 | 33.9 | 35.0 | 36.2 | 37.5 | 39.0 | 40.8 | 43.4 |
| 10.0-10.9 | BCPE | 16.8 | 19.9 | 22.0 | 23.6 | 25.0 | 26.3 | 27.5 | 28.7 | 29.8 | 30.8 | 31.9 | 33.0 | 34.1 | 35.2 | 36.4 | 37.7 | 39.2 | 41.0 | 43.6 |
| 11.0-11.9 | BCPE | 17.0 | 20.1 | 22.2 | 23.9 | 25.3 | 26.6 | 27.8 | 28.9 | 30.1 | 31.2 | 32.2 | 33.3 | 34.4 | 35.6 | 36.8 | 38.1 | 39.6 | 41.5 | 44.1 |
| 12.0-12.9 | BCPE | 17.3 | 20.4 | 22.6 | 24.3 | 25.7 | 27.0 | 28.3 | 29.4 | 30.6 | 31.7 | 32.8 | 33.9 | 35.0 | 36.2 | 37.4 | 38.8 | 40.3 | 42.2 | 44.8 |

BCPE: Box-Cox power exponential distribution; GAMLSS: generalized additive models for location, scale, and shape; NO: normal distribution

**Table 3.** Percentiles of total handgrip strength (kg), by gender and age, calculated with generalized additive models for location, scale, and shape (GAMLSS).

| Gender/Age | GAMLSS | Percentiles | | | | | | | | | | | | | | | | | | |
| --- | --- | --- | --- | --- | --- | --- | --- | --- | --- | --- | --- | --- | --- | --- | --- | --- | --- | --- | --- | --- |
|  |  | 5 | 10 | 15 | 20 | 25 | 30 | 35 | 40 | 45 | 50 | 55 | 60 | 65 | 70 | 75 | 80 | 85 | 90 | 95 |
| Boys |  |  |  |  |  |  |  |  |  |  |  |  |  |  |  |  |  |  |  |  |
| 8.0-8.9 | BCPE | 16.2 | 18.1 | 19.4 | 20.5 | 21.5 | 22.3 | 23.1 | 23.9 | 24.6 | 25.4 | 26.1 | 26.9 | 27.7 | 28.6 | 29.6 | 30.8 | 32.1 | 33.9 | 36.7 |
| 9.0-9.9 | BCPE | 18.3 | 20.4 | 21.9 | 23.2 | 24.2 | 25.2 | 26.1 | 27.0 | 27.8 | 28.7 | 29.5 | 30.4 | 31.3 | 32.3 | 33.5 | 34.7 | 36.3 | 38.3 | 41.4 |
| 10.0-10.9 | BCPE | 20.1 | 22.5 | 24.2 | 25.6 | 26.7 | 27.8 | 28.8 | 29.8 | 30.7 | 31.6 | 32.6 | 33.5 | 34.6 | 35.7 | 36.9 | 38.3 | 40.0 | 42.3 | 45.7 |
| 11.0-11.9 | BCPE | 22.0 | 24.7 | 26.5 | 28.0 | 29.3 | 30.4 | 31.5 | 32.6 | 33.6 | 34.6 | 35.6 | 36.7 | 37.8 | 39.0 | 40.4 | 41.9 | 43.8 | 46.2 | 50.0 |
| 12.0-12.9 | BCPE | 25.3 | 28.4 | 30.5 | 32.2 | 33.6 | 35.0 | 36.2 | 37.4 | 38.6 | 39.8 | 41.0 | 42.2 | 43.5 | 44.9 | 46.4 | 48.2 | 50.4 | 53.1 | 57.5 |
| Girls |  |  |  |  |  |  |  |  |  |  |  |  |  |  |  |  |  |  |  |  |
| 8.0-8.9 | BCPE | 15.4 | 17.2 | 18.5 | 19.5 | 20.4 | 21.2 | 22.0 | 22.7 | 23.4 | 24.1 | 24.8 | 25.5 | 26.3 | 27.2 | 28.1 | 29.2 | 30.5 | 32.2 | 34.8 |
| 9.0-9.9 | BCPE | 17.0 | 19.0 | 20.4 | 21.5 | 22.5 | 23.4 | 24.2 | 25.0 | 25.8 | 26.5 | 27.3 | 28.1 | 29.0 | 29.9 | 30.9 | 32.1 | 33.5 | 35.4 | 38.3 |
| 10.0-10.9 | BCPE | 18.7 | 20.9 | 22.5 | 23.7 | 24.8 | 25.7 | 26.6 | 27.5 | 28.4 | 29.2 | 30.1 | 31.0 | 31.9 | 32.9 | 34.1 | 35.4 | 37.0 | 39.0 | 42.2 |
| 11.0-11.9 | BCPE | 21.1 | 23.5 | 25.3 | 26.6 | 27.9 | 29.0 | 30.0 | 31.0 | 31.9 | 32.9 | 33.8 | 34.8 | 35.9 | 37.1 | 38.3 | 39.8 | 41.6 | 43.9 | 47.5 |
| 12.0-12.9 | BCPE | 24.2 | 27.1 | 29.0 | 30.6 | 32.0 | 33.3 | 34.5 | 35.6 | 36.7 | 37.8 | 38.9 | 40.1 | 41.3 | 42.6 | 44.1 | 45.8 | 47.8 | 50.4 | 54.6 |

BCPE: Box-Cox power exponential distribution; GAMLSS: generalized additive models for location, scale, and shape

**Table 4.** Percentiles of the PACER (20-metre laps), by gender and age, calculated with generalized additive models for location, scale, and shape (GAMLSS).

| Gender/Age | GAMLSS | Percentiles | | | | | | | | | | | | | | | | | | |
| --- | --- | --- | --- | --- | --- | --- | --- | --- | --- | --- | --- | --- | --- | --- | --- | --- | --- | --- | --- | --- |
|  |  | 5 | 10 | 15 | 20 | 25 | 30 | 35 | 40 | 45 | 50 | 55 | 60 | 65 | 70 | 75 | 80 | 85 | 90 | 95 |
| Boys |  |  |  |  |  |  |  |  |  |  |  |  |  |  |  |  |  |  |  |  |
| 8.0-8.9 | BCPE | 6 | 8 | 9 | 11 | 12 | 13 | 15 | 16 | 18 | 19 | 21 | 23 | 25 | 28 | 30 | 33 | 37 | 41 | 48 |
| 9.0-9.9 | BCPE | 7 | 8 | 10 | 11 | 13 | 14 | 16 | 17 | 19 | 21 | 23 | 25 | 27 | 29 | 32 | 35 | 39 | 44 | 51 |
| 10.0-10.9 | BCPE | 7 | 9 | 10 | 12 | 13 | 15 | 16 | 18 | 20 | 21 | 23 | 26 | 28 | 31 | 33 | 37 | 41 | 45 | 53 |
| 11.0-11.9 | BCPE | 7 | 9 | 11 | 12 | 14 | 15 | 17 | 19 | 21 | 23 | 25 | 27 | 30 | 32 | 35 | 39 | 43 | 48 | 56 |
| 12.0-12.9 | BCPE | 8 | 10 | 12 | 14 | 15 | 17 | 19 | 21 | 23 | 25 | 28 | 30 | 33 | 36 | 39 | 43 | 48 | 54 | 63 |
| Girls |  |  |  |  |  |  |  |  |  |  |  |  |  |  |  |  |  |  |  |  |
| 8.0-8.9 | BCPE | 6 | 8 | 9 | 10 | 11 | 12 | 13 | 14 | 15 | 16 | 17 | 18 | 19 | 21 | 23 | 25 | 27 | 31 | 37 |
| 9.0-9.9 | BCPE | 7 | 8 | 9 | 10 | 11 | 12 | 13 | 14 | 16 | 17 | 18 | 19 | 21 | 22 | 24 | 26 | 29 | 33 | 39 |
| 10.0-10.9 | BCPE | 7 | 9 | 10 | 11 | 12 | 13 | 14 | 15 | 16 | 17 | 18 | 20 | 21 | 23 | 25 | 27 | 30 | 34 | 40 |
| 11.0-11.9 | BCPE | 8 | 9 | 10 | 12 | 13 | 14 | 15 | 16 | 17 | 19 | 20 | 21 | 23 | 25 | 27 | 29 | 32 | 36 | 43 |
| 12.0-12.9 | BCPE | 8 | 10 | 12 | 13 | 14 | 15 | 17 | 18 | 19 | 21 | 22 | 24 | 26 | 28 | 30 | 33 | 36 | 41 | 48 |

BCPE: Box-Cox power exponential distribution; GAMLSS: generalized additive models for location, scale, and shape; PACER: Progressive Aerobic Cardiovascular Endurance Run

**Table 5.** Percentiles of plank time (seconds), by gender and age, calculated with generalized additive models for location, scale, and shape (GAMLSS).

| Gender/Age | GAMLSS | Percentiles | | | | | | | | | | | | | | | | | | |
| --- | --- | --- | --- | --- | --- | --- | --- | --- | --- | --- | --- | --- | --- | --- | --- | --- | --- | --- | --- | --- |
|  |  | 5 | 10 | 15 | 20 | 25 | 30 | 35 | 40 | 45 | 50 | 55 | 60 | 65 | 70 | 75 | 80 | 85 | 90 | 95 |
| Boys |  |  |  |  |  |  |  |  |  |  |  |  |  |  |  |  |  |  |  |  |
| 8.0-8.9 | NO | 12.1 | 17.0 | 21.1 | 24.7 | 28.1 | 31.4 | 34.7 | 38.0 | 41.4 | 44.9 | 48.7 | 52.8 | 57.5 | 62.8 | 69.2 | 77.0 | 87.4 | 102.3 | 129.2 |
| 9.0-9.9 | NO | 12.5 | 17.6 | 21.8 | 25.5 | 29.1 | 32.5 | 35.9 | 39.3 | 42.8 | 46.4 | 50.3 | 54.6 | 59.4 | 64.9 | 71.5 | 79.6 | 90.2 | 105.7 | 133.4 |
| 10.0-10.9 | NO | 13.8 | 19.4 | 24.0 | 28.2 | 32.1 | 35.9 | 39.7 | 43.4 | 47.3 | 51.3 | 55.6 | 60.3 | 65.6 | 71.7 | 79.0 | 88.0 | 99.7 | 116.8 | 147.5 |
| 11.0-11.9 | NO | 13.4 | 18.8 | 23.3 | 27.3 | 31.1 | 34.8 | 38.4 | 42.1 | 45.8 | 49.7 | 53.9 | 58.4 | 63.6 | 69.5 | 76.5 | 85.2 | 96.6 | 113.1 | 142.8 |
| 12.0-12.9 | NO | 15.4 | 21.7 | 26.8 | 31.4 | 35.8 | 40.0 | 44.2 | 48.4 | 52.7 | 57.2 | 62.0 | 67.2 | 73.1 | 80.0 | 88.0 | 98.0 | 111.1 | 130.1 | 164.3 |
| Girls |  |  |  |  |  |  |  |  |  |  |  |  |  |  |  |  |  |  |  |  |
| 8.0-8.9 | BCPE | 13.6 | 18.7 | 22.9 | 26.5 | 30.0 | 33.4 | 36.7 | 40.0 | 43.4 | 46.9 | 50.6 | 54.8 | 59.4 | 64.7 | 71.1 | 78.9 | 89.3 | 104.3 | 131.4 |
| 9.0-9.9 | BCPE | 14.1 | 19.4 | 23.6 | 27.4 | 31.0 | 34.5 | 37.9 | 41.3 | 44.8 | 48.4 | 52.3 | 56.6 | 61.4 | 66.9 | 73.5 | 81.6 | 92.2 | 107.8 | 135.8 |
| 10.0-10.9 | BCPE | 14.6 | 20.0 | 24.4 | 28.3 | 32.0 | 35.6 | 39.1 | 42.6 | 46.3 | 50.0 | 54.0 | 58.4 | 63.4 | 69.1 | 75.8 | 84.2 | 95.2 | 111.2 | 140.1 |
| 11.0-11.9 | BCPE | 15.0 | 20.6 | 25.1 | 29.2 | 33.0 | 36.7 | 40.3 | 44.0 | 47.7 | 51.6 | 55.7 | 60.2 | 65.3 | 71.2 | 78.2 | 86.8 | 98.2 | 114.7 | 144.5 |
| 12.0-12.9 | BCPE | 15.5 | 21.2 | 25.9 | 30.1 | 34.0 | 37.8 | 41.5 | 45.3 | 49.1 | 53.1 | 57.4 | 62.1 | 67.3 | 73.4 | 80.6 | 89.5 | 101.2 | 118.2 | 148.9 |

BCPE: Box-Cox power exponential distribution; GAMLSS: generalized additive models for location, scale, and shape; NO: normal distribution

**Table 6.** Percentiles of waist circumference (cm), by gender and age, calculated with generalized additive models for location, scale, and shape (GAMLSS).

| Gender/Age | GAMLSS | Percentiles | | | | | | | | | | | | | | | | | | |
| --- | --- | --- | --- | --- | --- | --- | --- | --- | --- | --- | --- | --- | --- | --- | --- | --- | --- | --- | --- | --- |
|  |  | 5 | 10 | 15 | 20 | 25 | 30 | 35 | 40 | 45 | 50 | 55 | 60 | 65 | 70 | 75 | 80 | 85 | 90 | 95 |
| Boys |  |  |  |  |  |  |  |  |  |  |  |  |  |  |  |  |  |  |  |  |
| 8.0-8.9 | BCPE | 50.2 | 51.8 | 53.0 | 54.0 | 55.0 | 55.9 | 56.8 | 57.7 | 58.6 | 59.6 | 60.7 | 61.8 | 63.0 | 64.4 | 65.9 | 67.8 | 70.2 | 73.6 | 79.6 |
| 9.0-9.9 | BCPE | 52.1 | 53.7 | 54.9 | 56.0 | 57.0 | 57.9 | 58.9 | 59.8 | 60.8 | 61.8 | 62.9 | 64.1 | 65.3 | 66.7 | 68.3 | 70.3 | 72.8 | 76.3 | 82.5 |
| 10.0-10.9 | BCPE | 53.9 | 55.6 | 56.9 | 58.0 | 59.0 | 60.0 | 61.0 | 61.9 | 63.0 | 64.0 | 65.1 | 66.3 | 67.6 | 69.1 | 70.8 | 72.8 | 75.3 | 79.0 | 85.4 |
| 11.0-11.9 | BCPE | 55.8 | 57.5 | 58.8 | 60.0 | 61.0 | 62.0 | 63.1 | 64.1 | 65.1 | 66.2 | 67.4 | 68.6 | 70.0 | 71.5 | 73.2 | 75.3 | 77.9 | 81.7 | 88.4 |
| 12.0-12.9 | BCPE | 57.6 | 59.4 | 60.8 | 62.0 | 63.1 | 64.1 | 65.1 | 66.2 | 67.3 | 68.4 | 69.6 | 70.9 | 72.3 | 73.8 | 75.6 | 77.8 | 80.5 | 84.4 | 91.3 |
| Girls |  |  |  |  |  |  |  |  |  |  |  |  |  |  |  |  |  |  |  |  |
| 8.0-8.9 | BCPE | 49.5 | 51.2 | 52.4 | 53.5 | 54.6 | 55.6 | 56.6 | 57.6 | 58.7 | 59.8 | 61.0 | 62.2 | 63.5 | 65.0 | 66.6 | 68.4 | 70.7 | 73.7 | 78.6 |
| 9.0-9.9 | BCPE | 51.3 | 53.0 | 54.3 | 55.5 | 56.6 | 57.6 | 58.7 | 59.7 | 60.8 | 62.0 | 63.2 | 64.5 | 65.8 | 67.3 | 69.0 | 70.9 | 73.3 | 76.4 | 81.5 |
| 10.0-10.9 | BCPE | 53.2 | 54.9 | 56.3 | 57.5 | 58.6 | 59.6 | 60.7 | 61.8 | 63.0 | 64.2 | 65.4 | 66.7 | 68.2 | 69.7 | 71.4 | 73.4 | 75.9 | 79.1 | 84.4 |
| 11.0-11.9 | BCPE | 55.0 | 56.8 | 58.2 | 59.4 | 60.6 | 61.7 | 62.8 | 64.0 | 65.1 | 66.4 | 67.7 | 69.0 | 70.5 | 72.1 | 73.9 | 76.0 | 78.5 | 81.8 | 87.3 |
| 12.0-12.9 | BCPE | 56.8 | 58.7 | 60.2 | 61.4 | 62.6 | 63.8 | 64.9 | 66.1 | 67.3 | 68.6 | 69.9 | 71.4 | 72.9 | 74.5 | 76.4 | 78.5 | 81.1 | 84.6 | 90.2 |

BCPE: Box-Cox power exponential distribution; GAMLSS: generalized additive models for location, scale, and shape

**Table 7.** Percentiles of the CAMSA max score (out of 28), by gender and age, calculated with generalized additive models for location, scale, and shape (GAMLSS).

| Gender/Age | GAMLSS | Percentiles | | | | | | | | | | | | | | | | | | |
| --- | --- | --- | --- | --- | --- | --- | --- | --- | --- | --- | --- | --- | --- | --- | --- | --- | --- | --- | --- | --- |
|  |  | 5 | 10 | 15 | 20 | 25 | 30 | 35 | 40 | 45 | 50 | 55 | 60 | 65 | 70 | 75 | 80 | 85 | 90 | 95 |
| Boys |  |  |  |  |  |  |  |  |  |  |  |  |  |  |  |  |  |  |  |  |
| 8.0-8.9 | BCCG | 13 | 15 | 16 | 17 | 17 | 18 | 19 | 19 | 19 | 20 | 20 | 21 | 21 | 21 | 22 | 22 | 23 | 23 | 24 |
| 9.0-9.9 | BCCG | 13 | 15 | 16 | 17 | 18 | 19 | 19 | 20 | 20 | 21 | 21 | 21 | 22 | 22 | 23 | 23 | 23 | 24 | 25 |
| 10.0-10.9 | BCCG | 13 | 15 | 17 | 18 | 19 | 19 | 20 | 20 | 21 | 21 | 22 | 22 | 22 | 23 | 23 | 24 | 24 | 25 | 26 |
| 11.0-11.9 | BCCG | 14 | 16 | 17 | 18 | 19 | 20 | 20 | 21 | 21 | 22 | 22 | 23 | 23 | 24 | 24 | 25 | 25 | 26 | 26 |
| 12.0-12.9 | BCCG | 14 | 16 | 18 | 19 | 20 | 20 | 21 | 22 | 22 | 23 | 23 | 23 | 24 | 24 | 25 | 25 | 26 | 26 | 27 |
| Girls |  |  |  |  |  |  |  |  |  |  |  |  |  |  |  |  |  |  |  |  |
| 8.0-8.9 | BCPE | 12 | 14 | 15 | 15 | 16 | 17 | 17 | 18 | 18 | 18 | 19 | 19 | 20 | 20 | 20 | 21 | 21 | 22 | 23 |
| 9.0-9.9 | BCPE | 13 | 15 | 16 | 16 | 17 | 17 | 18 | 18 | 19 | 19 | 20 | 20 | 21 | 21 | 21 | 22 | 22 | 23 | 24 |
| 10.0-10.9 | BCPE | 14 | 15 | 16 | 17 | 18 | 18 | 19 | 19 | 20 | 20 | 21 | 21 | 22 | 22 | 22 | 23 | 23 | 24 | 25 |
| 11.0-11.9 | BCPE | 14 | 16 | 17 | 18 | 18 | 19 | 19 | 20 | 21 | 21 | 21 | 22 | 22 | 23 | 23 | 24 | 24 | 25 | 26 |
| 12.0-12.9 | BCPE | 15 | 16 | 17 | 18 | 19 | 20 | 20 | 21 | 21 | 22 | 22 | 23 | 23 | 24 | 24 | 25 | 25 | 26 | 27 |

BCCG: Box-Cox Cole and Green distribution**;** BCPE: Box-Cox power exponential distribution; CAMSA: Canadian Agility and Movement Skill Assessment; GAMLSS - generalized additive models for location, scale, and shape

**Table 8.** Percentiles of the Daily Behaviour score (out of 32), by gender and age, calculated with generalized additive models for location, scale, and shape (GAMLSS).

| Gender/Age | GAMLSS | Percentiles | | | | | | | | | | | | | | | | | | |
| --- | --- | --- | --- | --- | --- | --- | --- | --- | --- | --- | --- | --- | --- | --- | --- | --- | --- | --- | --- | --- |
|  |  | 5 | 10 | 15 | 20 | 25 | 30 | 35 | 40 | 45 | 50 | 55 | 60 | 65 | 70 | 75 | 80 | 85 | 90 | 95 |
| Boys |  |  |  |  |  |  |  |  |  |  |  |  |  |  |  |  |  |  |  |  |
| 8.0-8.9 | BCPE | 4.2 | 6.6 | 8.7 | 10.5 | 12.3 | 13.9 | 15.5 | 17.0 | 19.8 | 19.8 | 19.8 | 22.5 | 23.8 | 25.0 | 26.3 | 27.5 | 28.7 | 29.8 | 31.0 |
| 9.0-9.9 | BCPE | 4.2 | 6.6 | 8.7 | 10.5 | 12.3 | 13.9 | 15.5 | 17.0 | 19.8 | 19.8 | 19.8 | 22.5 | 23.8 | 25.1 | 26.3 | 27.5 | 28.7 | 29.9 | 31.0 |
| 10.0-10.9 | BCPE | 4.2 | 6.6 | 8.7 | 10.5 | 12.3 | 13.9 | 15.5 | 17.0 | 19.8 | 19.8 | 19.8 | 22.5 | 23.8 | 25.0 | 26.3 | 27.5 | 28.7 | 29.8 | 31.0 |
| 11.0-11.9 | BCPE | 4.2 | 6.6 | 8.7 | 10.5 | 12.3 | 13.9 | 15.5 | 17.0 | 19.8 | 19.8 | 19.8 | 22.5 | 23.8 | 25.0 | 26.3 | 27.5 | 28.7 | 29.8 | 31.0 |
| 12.0-12.9 | BCPE | 4.2 | 6.6 | 8.7 | 10.5 | 12.3 | 13.9 | 15.5 | 17.0 | 19.8 | 19.8 | 19.8 | 22.5 | 23.8 | 25.0 | 26.3 | 27.5 | 28.7 | 29.8 | 31.0 |
| Girls |  |  |  |  |  |  |  |  |  |  |  |  |  |  |  |  |  |  |  |  |
| 8.0-8.9 | BCPE | 7.0 | 9.2 | 10.9 | 12.3 | 13.6 | 14.8 | 16.0 | 17.2 | 18.4 | 19.5 | 20.7 | 21.9 | 23.0 | 24.2 | 25.4 | 26.6 | 27.9 | 29.5 | 31.5 |
| 9.0-9.9 | BCPE | 7.0 | 9.2 | 10.8 | 12.3 | 13.5 | 14.8 | 16.0 | 17.1 | 18.3 | 19.5 | 20.6 | 21.8 | 22.9 | 24.1 | 25.3 | 26.5 | 27.8 | 29.4 | 31.4 |
| 10.0-10.9 | BCPE | 6.9 | 9.0 | 10.7 | 12.0 | 13.3 | 14.5 | 15.7 | 16.8 | 18.0 | 19.1 | 20.3 | 21.4 | 22.5 | 23.7 | 24.8 | 26.0 | 27.4 | 28.9 | 30.9 |
| 11.0-11.9 | BCPE | 6.6 | 8.7 | 10.3 | 11.6 | 12.9 | 14.0 | 15.2 | 16.3 | 17.4 | 18.5 | 19.6 | 20.7 | 21.8 | 22.9 | 24.0 | 25.2 | 26.4 | 27.9 | 29.8 |
| 12.0-12.9 | BCPE | 6.6 | 8.7 | 10.3 | 11.6 | 12.8 | 14.0 | 15.1 | 16.2 | 17.3 | 18.4 | 19.5 | 20.6 | 21.7 | 22.8 | 23.9 | 25.1 | 26.3 | 27.8 | 29.7 |

BCPE: Box-Cox power exponential distribution; GAMLSS: generalized additive models for location, scale, and shape

**Table 9.** Percentiles of daily steps taken, by gender and age, calculated with generalized additive models for location, scale, and shape (GAMLSS).

| Gender/Age | GAMLSS | Percentiles | | | | | | | | | | | | | | | | | | |
| --- | --- | --- | --- | --- | --- | --- | --- | --- | --- | --- | --- | --- | --- | --- | --- | --- | --- | --- | --- | --- |
|  |  | 5 | 10 | 15 | 20 | 25 | 30 | 35 | 40 | 45 | 50 | 55 | 60 | 65 | 70 | 75 | 80 | 85 | 90 | 95 |
| Boys |  |  |  |  |  |  |  |  |  |  |  |  |  |  |  |  |  |  |  |  |
| 8.0-8.9 | BCCG | 6353 | 7646 | 8572 | 9337 | 10015 | 10639 | 11230 | 11803 | 12368 | 12934 | 13511 | 14107 | 14733 | 15405 | 16143 | 16982 | 17980 | 19268 | 21241 |
| 9.0-9.9 | BCCG | 6183 | 7442 | 8343 | 9088 | 9747 | 10354 | 10930 | 11488 | 12038 | 12589 | 13150 | 13730 | 14340 | 14994 | 15712 | 16528 | 17500 | 18753 | 20674 |
| 10.0-10.9 | BCCG | 6014 | 7238 | 8114 | 8839 | 9480 | 10070 | 10630 | 11173 | 11708 | 12244 | 12789 | 13353 | 13946 | 14582 | 15281 | 16075 | 17020 | 18239 | 20107 |
| 11.0-11.9 | BCCG | 5844 | 7034 | 7885 | 8589 | 9212 | 9786 | 10330 | 10858 | 11377 | 11898 | 12429 | 12977 | 13553 | 14171 | 14850 | 15621 | 16539 | 17724 | 19539 |
| 12.0-12.9 | BCCG | 5674 | 6830 | 7656 | 8340 | 8945 | 9502 | 10031 | 10542 | 11047 | 11553 | 12068 | 12600 | 13160 | 13760 | 14419 | 15168 | 16059 | 17210 | 18972 |
| Girls |  |  |  |  |  |  |  |  |  |  |  |  |  |  |  |  |  |  |  |  |
| 8.0-8.9 | BCPE | 5996 | 7047 | 7799 | 8420 | 8968 | 9472 | 9949 | 10409 | 10862 | 11316 | 11781 | 12269 | 12790 | 13360 | 13998 | 14739 | 15643 | 16845 | 18763 |
| 9.0-9.9 | BCPE | 5814 | 6833 | 7562 | 8164 | 8696 | 9184 | 9646 | 10093 | 10532 | 10972 | 11423 | 11896 | 12402 | 12954 | 13573 | 14291 | 15168 | 16334 | 18193 |
| 10.0-10.9 | BCPE | 5632 | 6618 | 7325 | 7908 | 8423 | 8896 | 9344 | 9776 | 10202 | 10628 | 11065 | 11523 | 12013 | 12548 | 13147 | 13843 | 14692 | 15822 | 17623 |
| 11.0-11.9 | BCPE | 5450 | 6404 | 7088 | 7652 | 8151 | 8609 | 9042 | 9460 | 9872 | 10284 | 10707 | 11151 | 11624 | 12142 | 12722 | 13395 | 14217 | 15310 | 17053 |
| 12.0-12.9 | BCPE | 5267 | 6190 | 6851 | 7396 | 7878 | 8321 | 8739 | 9144 | 9542 | 9941 | 10349 | 10778 | 11236 | 11736 | 12296 | 12947 | 13742 | 14798 | 16482 |

BCCG: Box-Cox Cole and Green distribution; BCPE: Box-Cox power exponential distribution; GAMLSS: generalized additive models for location, scale, and shape

**Table 10.** Percentiles of the Motivation and Confidence score (out of 18), by gender and age, calculated with generalized additive models for location, scale, and shape (GAMLSS).

| Gender/Age | GAMLSS | Percentiles | | | | | | | | | | | | | | | | | | |
| --- | --- | --- | --- | --- | --- | --- | --- | --- | --- | --- | --- | --- | --- | --- | --- | --- | --- | --- | --- | --- |
|  |  | 5 | 10 | 15 | 20 | 25 | 30 | 35 | 40 | 45 | 50 | 55 | 60 | 65 | 70 | 75 | 80 | 85 | 90 | 95 |
| Boys |  |  |  |  |  |  |  |  |  |  |  |  |  |  |  |  |  |  |  |  |
| 8.0-8.9 | BCPE | 7.4 | 8.7 | 9.5 | 10.2 | 10.7 | 11.2 | 11.6 | 12.0 | 12.4 | 12.7 | 13.1 | 13.4 | 13.8 | 14.1 | 14.4 | 14.8 | 15.2 | 15.6 | 16.2 |
| 9.0-9.9 | BCPE | 7.5 | 8.9 | 9.7 | 10.3 | 10.9 | 11.3 | 11.8 | 12.2 | 12.6 | 12.9 | 13.3 | 13.6 | 14.0 | 14.3 | 14.7 | 15.0 | 15.4 | 15.8 | 16.4 |
| 10.0-10.9 | BCPE | 7.6 | 9.0 | 9.8 | 10.4 | 11.0 | 11.5 | 11.9 | 12.3 | 12.7 | 13.1 | 13.4 | 13.8 | 14.1 | 14.5 | 14.8 | 15.2 | 15.6 | 16.0 | 16.6 |
| 11.0-11.9 | BCPE | 7.7 | 9.0 | 9.8 | 10.5 | 11.0 | 11.5 | 11.9 | 12.4 | 12.8 | 13.1 | 13.5 | 13.8 | 14.2 | 14.5 | 14.9 | 15.2 | 15.6 | 16.1 | 16.7 |
| 12.0-12.9 | BCPE | 7.7 | 9.0 | 9.9 | 10.5 | 11.1 | 11.5 | 12.0 | 12.4 | 12.8 | 13.2 | 13.5 | 13.9 | 14.2 | 14.6 | 14.9 | 15.3 | 15.7 | 16.1 | 16.7 |
| Girls |  |  |  |  |  |  |  |  |  |  |  |  |  |  |  |  |  |  |  |  |
| 8.0-8.9 | BCPE | 7.5 | 8.6 | 9.4 | 10.0 | 10.4 | 10.9 | 11.3 | 11.6 | 12.0 | 12.3 | 12.7 | 13.0 | 13.3 | 13.7 | 14.0 | 14.4 | 14.8 | 15.2 | 15.9 |
| 9.0-9.9 | BCPE | 7.6 | 8.7 | 9.5 | 10.1 | 10.6 | 11.0 | 11.4 | 11.8 | 12.1 | 12.5 | 12.8 | 13.2 | 13.5 | 13.8 | 14.2 | 14.5 | 14.9 | 15.4 | 16.0 |
| 10.0-10.9 | BCPE | 7.6 | 8.8 | 9.5 | 10.1 | 10.6 | 11.0 | 11.4 | 11.8 | 12.2 | 12.5 | 12.8 | 13.2 | 13.5 | 13.9 | 14.2 | 14.6 | 15.0 | 15.4 | 16.1 |
| 11.0-11.9 | BCPE | 7.6 | 8.7 | 9.4 | 10.0 | 10.5 | 10.9 | 11.3 | 11.7 | 12.1 | 12.4 | 12.8 | 13.1 | 13.4 | 13.8 | 14.1 | 14.5 | 14.9 | 15.3 | 16.0 |
| 12.0-12.9 | BCPE | 7.6 | 8.8 | 9.5 | 10.1 | 10.6 | 11.0 | 11.4 | 11.8 | 12.2 | 12.5 | 12.9 | 13.2 | 13.5 | 13.9 | 14.2 | 14.6 | 15.0 | 15.5 | 16.1 |

BCPE: Box-Cox power exponential distribution; GAMLSS: generalized additive models for location, scale, and shape

**Table 11.** Percentiles of the Knowledge and Understanding score (out of 18), by gender and age, calculated with generalized additive models for location, scale, and shape (GAMLSS).

| Gender/Age | GAMLSS | Percentiles | | | | | | | | | | | | | | | | | | |
| --- | --- | --- | --- | --- | --- | --- | --- | --- | --- | --- | --- | --- | --- | --- | --- | --- | --- | --- | --- | --- |
|  |  | 5 | 10 | 15 | 20 | 25 | 30 | 35 | 40 | 45 | 50 | 55 | 60 | 65 | 70 | 75 | 80 | 85 | 90 | 95 |
| Boys |  |  |  |  |  |  |  |  |  |  |  |  |  |  |  |  |  |  |  |  |
| 8.0-8.9 | BCPE | 6.2 | 7.2 | 7.8 | 8.3 | 8.8 | 9.2 | 9.5 | 9.9 | 10.2 | 10.6 | 10.9 | 11.2 | 11.6 | 11.9 | 12.2 | 12.5 | 12.9 | 13.3 | 13.9 |
| 9.0-9.9 | BCPE | 6.6 | 7.6 | 8.3 | 8.8 | 9.3 | 9.8 | 10.2 | 10.5 | 10.9 | 11.3 | 11.6 | 12.0 | 12.3 | 12.6 | 13.0 | 13.3 | 13.7 | 14.2 | 14.8 |
| 10.0-10.9 | BCPE | 6.9 | 8.0 | 8.7 | 9.3 | 9.8 | 10.3 | 10.7 | 11.1 | 11.5 | 11.9 | 12.2 | 12.6 | 13.0 | 13.3 | 13.7 | 14.1 | 14.5 | 14.9 | 15.6 |
| 11.0-11.9 | BCPE | 7.2 | 8.4 | 9.1 | 9.7 | 10.2 | 10.7 | 11.1 | 11.6 | 12.0 | 12.4 | 12.7 | 13.1 | 13.5 | 13.9 | 14.2 | 14.6 | 15.1 | 15.6 | 16.2 |
| 12.0-12.9 | BCPE | 7.4 | 8.5 | 9.3 | 9.9 | 10.4 | 10.9 | 11.3 | 11.8 | 12.2 | 12.6 | 13.0 | 13.4 | 13.7 | 14.1 | 14.5 | 14.9 | 15.3 | 15.8 | 16.5 |
| Girls |  |  |  |  |  |  |  |  |  |  |  |  |  |  |  |  |  |  |  |  |
| 8.0-8.9 | BCPE | 6.8 | 7.8 | 8.4 | 8.9 | 9.3 | 9.7 | 10.0 | 10.4 | 10.7 | 11.0 | 11.3 | 11.6 | 11.9 | 12.2 | 12.5 | 12.9 | 13.2 | 13.6 | 14.1 |
| 9.0-9.9 | BCPE | 7.2 | 8.1 | 8.8 | 9.3 | 9.8 | 10.2 | 10.5 | 10.9 | 11.2 | 11.6 | 11.9 | 12.2 | 12.5 | 12.8 | 13.2 | 13.5 | 13.9 | 14.3 | 14.8 |
| 10.0-10.9 | BCPE | 7.5 | 8.6 | 9.2 | 9.8 | 10.3 | 10.7 | 11.1 | 11.4 | 11.8 | 12.2 | 12.5 | 12.8 | 13.2 | 13.5 | 13.8 | 14.2 | 14.6 | 15.0 | 15.6 |
| 11.0-11.9 | BCPE | 7.8 | 8.9 | 9.6 | 10.2 | 10.7 | 11.1 | 11.5 | 11.9 | 12.3 | 12.7 | 13.0 | 13.4 | 13.7 | 14.0 | 14.4 | 14.8 | 15.1 | 15.6 | 16.2 |
| 12.0-12.9 | BCPE | 8.0 | 9.1 | 9.8 | 10.4 | 10.9 | 11.3 | 11.8 | 12.1 | 12.5 | 12.9 | 13.3 | 13.6 | 14.0 | 14.3 | 14.7 | 15.0 | 15.4 | 15.9 | 16.5 |

BCPE: Box-Cox power exponential distribution; GAMLSS: generalized additive models for location, scale, and shape

**Table 12.** Percentiles of the overall physical literacy score (out of 100), by gender and age, calculated with generalized additive models for location, scale, and shape (GAMLSS).

| Gender/Age | GAMLSS | Percentiles | | | | | | | | | | | | | | | | | | |
| --- | --- | --- | --- | --- | --- | --- | --- | --- | --- | --- | --- | --- | --- | --- | --- | --- | --- | --- | --- | --- |
|  |  | 5 | 10 | 15 | 20 | 25 | 30 | 35 | 40 | 45 | 50 | 55 | 60 | 65 | 70 | 75 | 80 | 85 | 90 | 95 |
| Boys |  |  |  |  |  |  |  |  |  |  |  |  |  |  |  |  |  |  |  |  |
| 8.0-8.9 | BCPE | 38.7 | 43.2 | 46.3 | 48.8 | 51.0 | 53.0 | 54.9 | 56.7 | 58.5 | 60.2 | 61.9 | 63.6 | 65.3 | 67.0 | 68.7 | 70.6 | 72.6 | 75.1 | 78.3 |
| 9.0-9.9 | BCPE | 39.9 | 44.6 | 47.8 | 50.3 | 52.6 | 54.7 | 56.6 | 58.5 | 60.3 | 62.1 | 63.9 | 65.6 | 67.4 | 69.1 | 70.9 | 72.9 | 75.0 | 77.5 | 80.8 |
| 10.0-10.9 | BCPE | 40.7 | 45.4 | 48.7 | 51.3 | 53.6 | 55.7 | 57.7 | 59.6 | 61.5 | 63.3 | 65.1 | 66.9 | 68.7 | 70.5 | 72.3 | 74.3 | 76.4 | 78.9 | 82.4 |
| 11.0-11.9 | BCPE | 41.0 | 45.8 | 49.1 | 51.8 | 54.1 | 56.2 | 58.2 | 60.2 | 62.0 | 63.9 | 65.7 | 67.5 | 69.3 | 71.1 | 72.9 | 74.9 | 77.1 | 79.6 | 83.1 |
| 12.0-12.9 | BCPE | 42.1 | 47.0 | 50.4 | 53.2 | 55.6 | 57.7 | 59.8 | 61.8 | 63.7 | 65.6 | 67.5 | 69.3 | 71.1 | 73.0 | 74.9 | 76.9 | 79.2 | 81.8 | 85.4 |
| Girls |  |  |  |  |  |  |  |  |  |  |  |  |  |  |  |  |  |  |  |  |
| 8.0-8.9 | BCPE | 41.8 | 45.9 | 48.7 | 50.9 | 52.8 | 54.5 | 56.1 | 57.6 | 59.0 | 60.5 | 61.9 | 63.3 | 64.8 | 66.3 | 67.9 | 69.7 | 71.7 | 74.1 | 77.6 |
| 9.0-9.9 | BCPE | 42.6 | 46.8 | 49.6 | 51.9 | 53.8 | 55.6 | 57.2 | 58.7 | 60.2 | 61.7 | 63.1 | 64.6 | 66.1 | 67.6 | 69.2 | 71.0 | 73.1 | 75.6 | 79.2 |
| 10.0-10.9 | BCPE | 43.1 | 47.4 | 50.2 | 52.5 | 54.4 | 56.2 | 57.8 | 59.4 | 60.9 | 62.4 | 63.8 | 65.3 | 66.8 | 68.4 | 70.0 | 71.8 | 73.9 | 76.4 | 80.1 |
| 11.0-11.9 | BCPE | 43.3 | 47.5 | 50.4 | 52.6 | 54.6 | 56.4 | 58.0 | 59.6 | 61.1 | 62.5 | 64.0 | 65.5 | 67.0 | 68.6 | 70.2 | 72.0 | 74.1 | 76.7 | 80.3 |
| 12.0-12.9 | BCPE | 43.9 | 48.3 | 51.2 | 53.5 | 55.5 | 57.2 | 58.9 | 60.5 | 62.0 | 63.5 | 65.0 | 66.5 | 68.1 | 69.7 | 71.3 | 73.2 | 75.3 | 77.9 | 81.6 |

BCPE: Box-Cox power exponential distribution; GAMLSS: generalized additive models for location, scale, and shape
